# Allele Frequencies at Recessive Disease Genes are Mainly Determined by Pleiotropic Effects in Heterozygotes

**DOI:** 10.64898/2025.12.05.692665

**Published:** 2025-12-09

**Authors:** Jonathan Judd, Jeffrey P. Spence, Nikhil Milind, Linda Kachuri, John S. Witte, Jonathan K. Pritchard

**Affiliations:** 1 Department of Genetics, Stanford University, Stanford, CA; 2 Institute for Human Genetics, University of California San Francisco, San Francisco, CA; 3 Department of Epidemiology & Biostatistics, University of California San Francisco, San Francisco, CA; 4 Department of Epidemiology and Population Health, Stanford University, Stanford, CA; 5 Stanford Cancer Institute, Stanford University School of Medicine, Stanford, CA; 6 Department of Biology, Stanford University, Stanford, CA

## Abstract

The classic theory of mutation-selection balance predicts the equilibrium frequency of genetic variation under negative selection. The model predicts a simple relationship between the total frequency of deleterious variants, mutation rate, and strength of selection, with different functions for recessive and (co-)dominant genes. In this study, we investigate whether genes associated with human recessive disorders fit the predictions of this classic model. By comparing observed frequencies of loss of function variants (LoFs) to those expected under mutation-selection balance we find that, for nearly all recessive genes, the observed frequencies are too low to be explained by purely recessive selection. Analyzing the effects of heterozygous LoFs on quantitative traits from the UK Biobank, we find that recessive disease genes have widespread quantitative effects in heterozygotes. Together, these results suggest that most selection experienced by pathogenic mutations in recessive disease genes may be due to stabilizing selection in heterozygotes. We conclude that very few human genes follow the classic model of recessive mutation-selection balance.

## Introduction

A century ago, J.B.S Haldane developed the now-classic theory of “mutation-selection balance”, which predicts the equilibrium frequency of deleterious variants [1, 2]. The key insight of mutation-selection balance is that, at equilibrium, the rate at which new deleterious variants are generated in a gene by mutations must exactly offset the rate at which variants are removed by natural selection.

In the last decade, advances in whole-exome and whole-genome sequencing have enabled sequencing of hundreds of thousands of healthy people [3–6]. Consistent with the predictions of mutation-selection balance, these studies show that important genes are highly depleted of functional variants such as loss-of-function (LoF) mutations [3–5]. This observation has motivated a great deal of work on estimating the strength of selection against LoF heterozygotes (here denoted $s_{\text{het}}$) for each gene, usually assuming additive models of selection [7–10].

However, the standard additive model does not apply to fully recessive genes. In this case, selection acts only in homozygotes; since homozygotes are rare compared to heterozygotes, the classic mutation selection-balance theory predicts that pathogenic variants should be found at much higher frequencies in recessive genes than in dominant or co-dominant genes.

It is generally believed that the recessive version of mutation-selection balance should apply to a wide variety of autosomal recessive diseases. Recessive diseases, including cystic fibrosis, sickle cell anemia, and Tay-Sachs disease [11–14], occur when patients inherit pathogenic mutations that abrogate function in both copies of a particular gene.

While significant progress has been made on estimating $s_{\text{het}}$, estimating selection coefficients in homozygotes (denoted $s_{\text{hom}}$) remains challenging due to the difficulty of disentangling heterozygous effects from homozgyous effects in population genetic studies, and due to the rarity of homozygotes and survivorship bias in observational studies [7, 15, 16].

Here, we take a different approach, simply asking whether the frequencies of putative pathogenic mutations in annotated recessive genes are compatible with recessive mutation-selection balance. After addressing several technical challenges we conclude that, for the vast majority of annotated recessive genes, the frequencies of pathogenic mutations are too low to be explained by a fully recessive model.

To explain this, we propose that most recessive disease genes also have meaningful phenotypic effects in heterozygotes — and that these pleiotropic effects are the main target of selection. At a mechanistic level, we can expect that heterozygous mutations would often reduce gene expression or functionality by 50%; this in turn might affect molecular phenotypes, such as chromatin accessibility, with cascading quantitative effects on organism-level traits [17–20]. Consistent with this prediction, we find that LoF mutations in genes annotated as being fully recessive frequently have significant effects on quantitative traits measured by UK Biobank. These heterozygous effects are likely too small to be recognized as clinical conditions, and yet strong enough to be subject to negative selection.

In summary, we report here that the frequencies of pathogenic mutations in nearly all annotated recessive genes are too low to be explained by Haldane's classical model of recessive mutation-selection balance. Instead we propose that most selection at these genes is due to stabilizing selection acting on quantitative effects in heterozygotes.

## Results

Mutation-selection balance predicts the total allele frequency of all pathogenic variants for a gene, $p_{\text{path}}$, by calculating the frequency at which the rate of new pathogenic alleles arising by mutation is exactly offset by the removal of alleles by natural selection:

(1) $$2\mu L=2p_{\text{path}}\left(1-p_{\text{path}}\right)s_{\text{het}}+2p_{\text{path}}^{2}s_{\text{hom}}$$

where μ denotes the per-base pair mutation rate, and L is the effective number of sites that can produce pathogenic mutations [1, 2]. When selection on heterozygotes, $s_{\text{het}}$, is non-negligible (i.e., for dominant or co-dominant traits), nearly all selection occurs in heterozygotes and the equilibrium frequency can be approximated as

(2) $$p_{\text{path}}=\frac{\mu L}{s_{\text{het}}};\;s_{\text{het}}\gg 0.$$

In contrast, for fully recessive genes, selection occurs only in homozygotes, so the equilibrium frequency is determined entirely by the frequency of homozygotes:

(3) $$p_{\text{path}}=\sqrt{\frac{\mu L}{s_{\text{hom}}}};\;s_{\text{het}}\approx 0.$$

**Figure 1:**
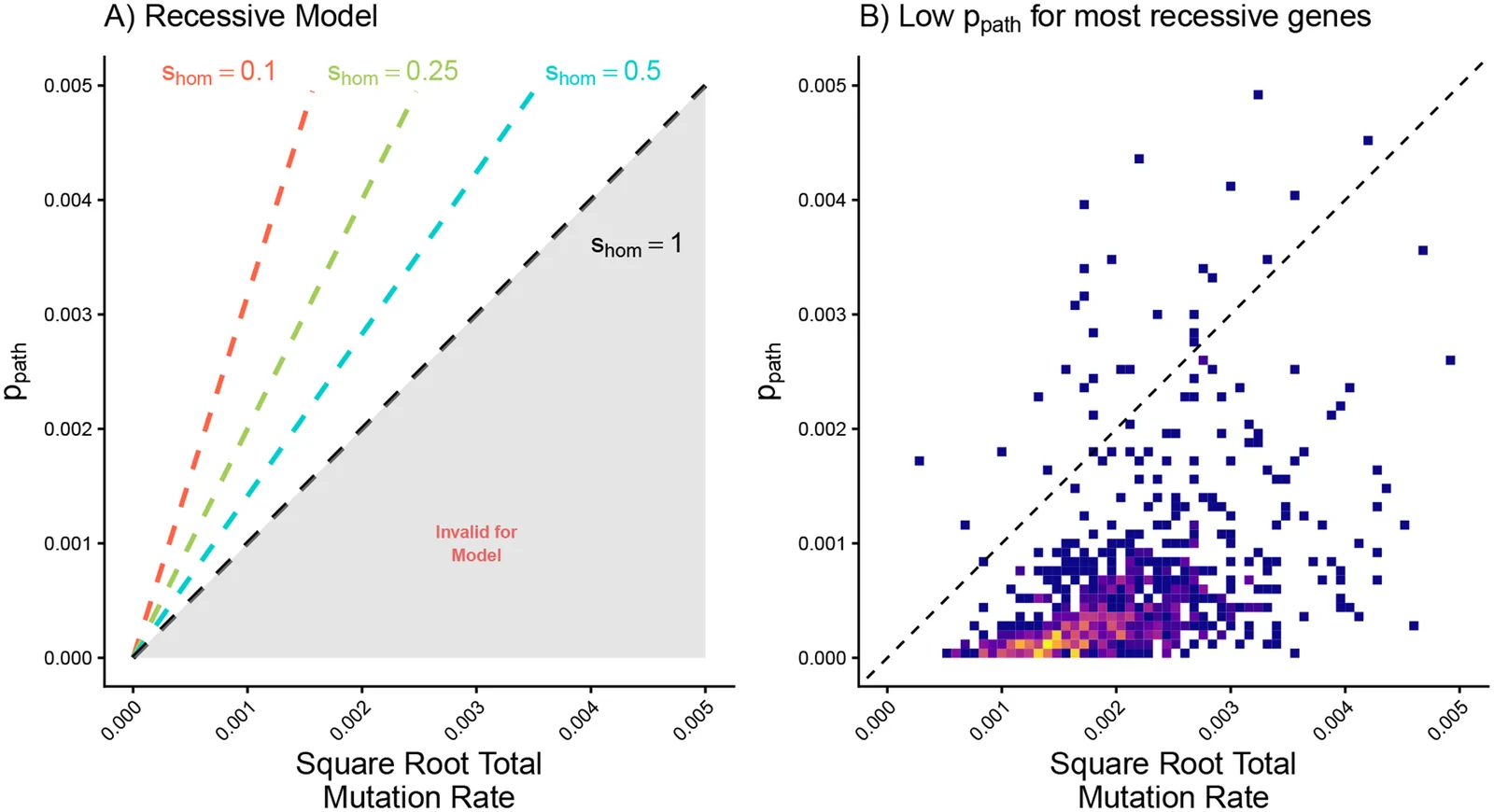
Observed Pathogenic Allele Frequency Versus Expected: **A)** Theoretical relationship between the square root mutation rate and $p_{\text{path}}$ under recessive mutation-selection balance, for a range of values of homozygous selection, $s_{\text{hom}}$. The shaded area represents parameter combinations that are incompatible with classic recessive mutation-selection balance. **B)** Density plot showing the observed relationship between square root total mutation rate and predicted $p_{\text{path}}$ for recessive genes. Most genes lie in the parameter region that is inconsistent with classic mutation-selection balance.

Under this model, the frequency of pathogenic alleles, $p_{\text{path}}$, is proportional to the square root of the gene's total mutation rate μL (Figure 1A). A key prediction for the present paper is that, even for fully lethal recessive mutations $\left(s_{\text{hom}}=1\right)$, selection cannot push the equilibrium frequency $p_{\text{path}}$ below $\sqrt{\mu L}$ for the corresponding gene.

Note that this classic derivation makes several simplifying assumptions that may not hold in practice (i.e., ignoring inbreeding, drift, and in-phase compound heterozygotes). We will consider these below.

### Curation of recessive disease genes.

To assess the extent to which purely recessive mutation-selection balance pertains to human genes, we began by curating a list of genes associated with only recessive diseases. To identify the most plausible purely recessive genes, we intersected the 19,194 protein-coding genes defined from the HUGO Gene Nomenclature Committee [21], with previous lists of recessive disease genes [16, 22, 23]. We then further filtered to remove genes with non-recessive effects reported in the Online Mendelian Inheritance in Man (OMIM) database [24]. This removes 387 genes, such as *CFTR* (cystic fibrosis) and *HBB* (sickle cell anemia), that are annotated as recessive, but also have documented heterozgyous or non-recessive effects.

This results in a final list of 796 genes with the strongest support for a fully recessive mode of inheritance (Table S1).

### Comparison of $p_{\text{path}}$ to model predictions.

Previous studies of selection coefficients in heterozygotes $\left(s_{\text{het}}\right)$ have generally focused on estimating the frequencies of certain types of loss-of-function mutations that can be accurately predicted computationally, and for which it is relatively straightforward to model the corresponding mutation rates. Specifically, recent studies have focused on annotating stop-gain, splice site donor-/acceptor-disrupting variants [5, 25].

Here, we refer to these specific categories of mutations as loss-of-function (LoF) mutations, and we denote the aggregate frequency of LoF variants as $p_{\text{LoF}}$. Gene-level information about LoF variants, including positions and allele frequencies, were retrieved from the gnomAD database v4.0, calculated separately for the Non-Finnish European (NFE), Finnish (FIN), African (AFR), and South Asian (SAS), ancestry groups [5, 25, 26]. We excluded variants with an allele frequency > 0.005 as previous work has shown that these are highly enriched for LoFs that are either bioinformatic errors, or may otherwise be functionally rescued [10]. (We show below that our qualitative results hold even without this filtering.)

We estimated the gene level mutation rate $\mu L_{\text{LOF}}$ (to LoFs) for each gene by counting all possible LoF-causing point mutations. We then computed a context-specific mutation rate that accounts for trinucleotide context and methylation status at CpGs for each possible LoF mutation; this was averaged across all L possible LoF-generating mutations, to estimate a total gene-level mutation rate [5, 25].

Initial analysis showed that, for nearly all recessive disease genes, the frequency of LoFs, $p_{\text{LoF}}$, lies far below the expected minimum frequency of $\sqrt{\mu L_{\text{LoF}}}$ indicating that $p_{\text{LoF}}$ cannot be predicted by mutation-selection balance under a purely recessive model (Figure S1A). However, this calculation fails to capture the fact that many individuals who are impacted by recessive disease are compound heterozygotes, carrying different mutations on each of their two gene copies. Thus, strictly speaking, the theory only applies to the full set of all possible pathogenic mutations, $p_{\text{path}}$.

Compared to annotating LoF mutations, it is much more difficult to confidently identify *all* pathogenic mutations for a given gene, let alone to estimate the total mutation rate that *could* generate pathogenic mutations.

As an alternative, we estimated a scaling factor that represents the fraction of all pathogenic mutations captured by LoFs. Specifically, we identified the molecular consequences of all known pathogenic variants for 25 well-studied recessive disease genes (Figure S1B) and then calculated the percentage of this variation that is captured by our annotated LoF mutations (Figure S1C, Table S2). We found that, on average, LoFs contributed around 32% of the total number of pathogenic variants. We then used the inverse of this fraction as a conversion factor to rescale $p_{\text{LoF}}$ and the LoF mutation rate $\mu L_{\text{LoF}}$ to estimate an overall $p_{\text{path}}$ and total mutation rate μL for each gene.

We were intrigued to find that, for the vast majority of recessive disease genes, the estimated $p_{\text{path}}$ is far lower than can be explained by the recessive mutation-selection balance model (Figure 1B). This result is observed when examining the relationship between $p_{\text{path}}$ and total mutation rate in recessive disease genes in other populations (Figure S2).

When the list of recessive genes is restricted to recessive-lethal genes, which are under the strongest homozgyous selection, we also see quantitatively similar results (Figure S3A).

Additional sensitivity analyses were performed to confirm that recessive disease gene selection and variant selection criteria, which might result in underestimating $p_{\text{path}}$, were not likely factors to explain these observations. We tested whether the data might fit if we removed the filter on unusually high-frequency LoFs (MAF > 0.005), but this makes little difference to the qualitative results (Figure S3B). We also wondered whether these observations might be explained if we are underestimating the ratio of LoFs to total pathogenic mutations. However, we find that we would need to increase this factor by around 104-fold to make 50% of genes fit the model and need to increase this factor an implausible amount (1,465-fold) to make 90% of genes fit the model.

To better understand why recessive disease genes are largely inconsistent with recessive mutation-selection balance, we next considered two potential categories of explanations: deviations from the basic model including inbreeding and drift, and unexpected quantitative effects in heterozygotes.

### Potential model deviations

The classic mutation-selection balance model makes three important simplifications that may not hold in practice: no inbreeding, no drift, and modeling the gene essentially as a single site with high mutation rate. We consider each of these in turn.

#### Inbreeding.

**Figure 2:**
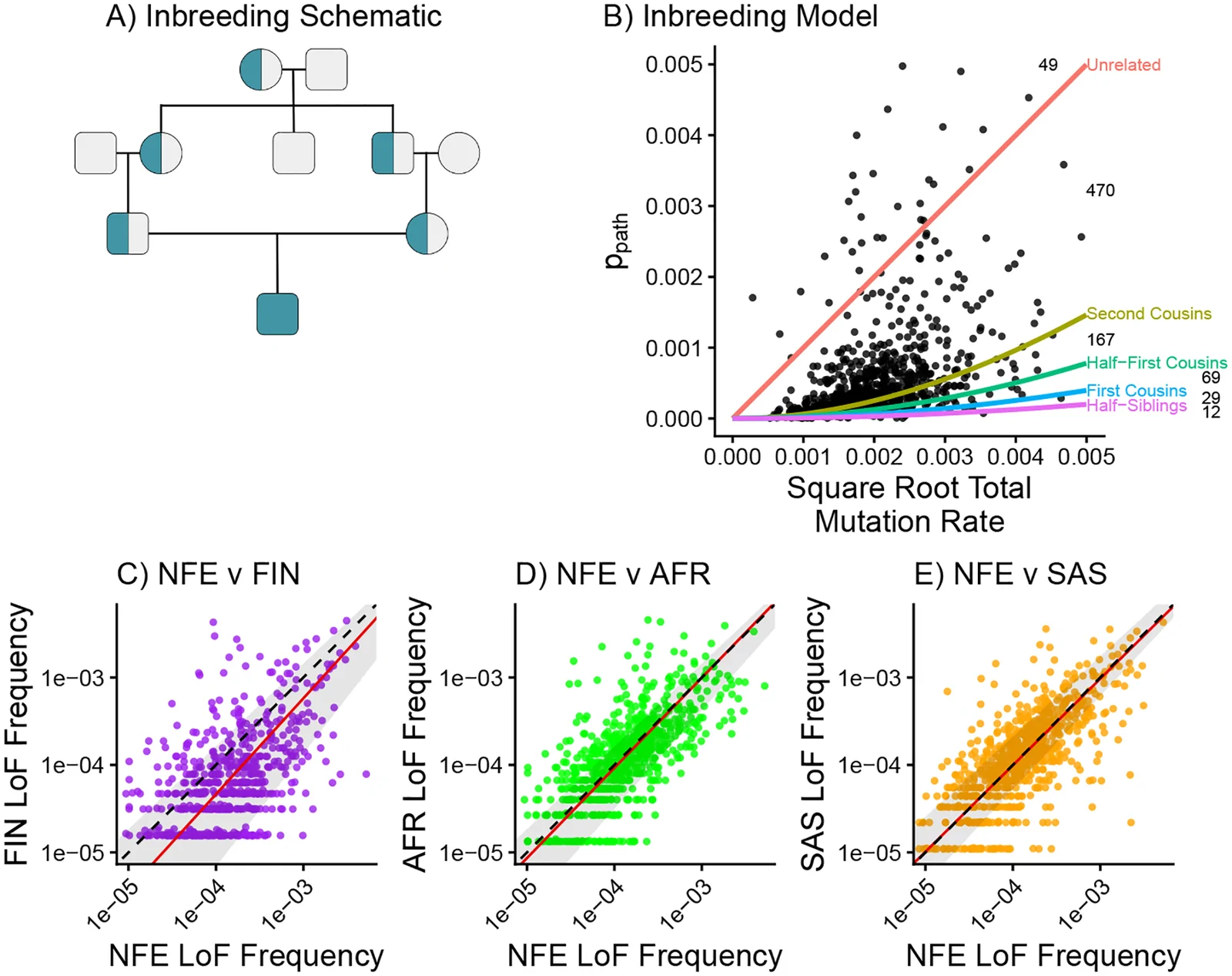
Inbreeding Under Mutation-Selection Balance: **A)** Illustration of how inbreeding, in this case between first cousins, increases the frequency of homozygotes in a population (conditional on $p_{\text{path}}$). **B)** The lines show the theoretical equilibrium $p_{\text{path}}$ under different levels of inbreeding; these are superimposed onto the observed data (e.g., dot is a single recessive gene). The numbers indicate the number of genes that lie between each adjacent pair of theoretical lines. Comparison of $p_{\text{LoF}}$ of recessive genes between NFE and **C)** Finnish (FIN) populations, **D)** African (AFR) populations, or **E)** South Asian (SAS) populations, respectively. Red line represents the total least squares regression of $p_{\text{LoF}}$ between populations. Shaded region represents 95% confidence interval of regression line. The black lines indicate y=x.

In our standard model, the probability that an individual is homozygous for pathogenic mutations follows Hardy-Weinberg expectations, i.e., $p_{\text{path}}^{2}$. However, many populations exhibit some degree of inbreeding — i.e., relatives mate with one another more often than expected under purely random mating. Close inbreeding dramatically increases the probability that an offspring inherits a pathogenic mutation from both parents (Figure 2A). Thus, conditional on allele frequency, inbreeding increases the probability that pathogenic mutations will be exposed to selection; this results in lower equilibrium frequencies compared to random mating [27]. We wanted to assess whether this effect is strong enough to explain our results.

To model this, we measure the extent of inbreeding using the probability that an individual inherits both of their copies of a gene identically-by-descent, which we denote φ. For example, under random mating φ=0; if 20% of matings are between first cousins then φ=0.0125. Under this model, mutation-selection balance takes the following form in the large population size limit:

(4) $$\underset{N\to \infty }{\text{lim}}E\left[p_{\text{path}}\right]=-\frac{\varphi }{2(1-\varphi )}+\sqrt{{\left(\frac{\varphi }{2(1-\varphi )}\right)}^{2}+\frac{\mu L}{(1-\varphi )s_{\text{hom}}}}$$

See Appendix A for details of the model and the derivation of the results.

Equation 4 highlights that higher inbreeding coefficients reduce the expected $p_{\text{path}}$ for a given total mutation rate. However, the extent of inbreeding would need to be implausibly high to explain the data: to explain 95% of recessive genes, we would need to assume that *all* matings occur between first cousins (Figure 2B, Figure S4A).

For comparison, some contemporary inbreeding rates (estimated from 1000 Genomes data [28]) identify low levels of second cousin inbreeding in non-Finnish European populations and no evidence of first-cousin inbreeding [29] while others (estimated from UK Biobank Data) similarily only identify low rates of second- and first-cousin inbreeding in specific European populations [30]. This indicates that inbreeding depression is unlikely to explain the failure of recessive mutation-selection balance to fit recessive disease genes.

#### Drift in small populations.

Equation 4 assumes an infinite-sized population, neglecting the effects of genetic drift on $p_{\text{path}}$. In contrast to the additive case, genetic drift lowers the expected $p_{\text{path}}$ under mutation-selection balance [31–34].

To address this, we derived an approximation that quantifies the effects of drift, inbreeding, and selection in homozygotes (Appendix A). Our approximation provides a good fit to forward-in-time Wright-Fisher simulations (Figure S4B). Under this adjusted model, inbreeding has a greater effect in reducing $p_{\text{path}}$, which enables more recessive disease genes to fit mutation-selection balance for lower levels of inbreeding. However, even when accounting for drift, fitting the data for most genes would still require inbreeding on the level of ubiquitous first cousin mating (Figure S4C, Figure S4D).

We also reasoned that if either of these demographic factors (inbreeding or drift) is somehow much larger than we expect, then it would likely vary in magnitude across populations. We would not expect that the precise parameters are the same in diverse populations. But when we analyze the fit for $p_{\text{path}}$ in diverse human populations we find only slight variation among populations and no clear bias (Figure 2C, Figure 2D, Figure 2E), a result previously described by Stolyarova *et al*. [26]. While there is evidence of a higher value of φ in South Asian (SAS) populations [29, 35], this does not result in the systematic reduction of $p_{\text{LoF}}$ relative to populations with lower levels φ that we would expect to see under a purely recessive model. The quantitative similarities among diverse populations suggest that a biological explanation is more likely than a demographic explanation.

#### In-phase compound heterozygotes.

Lastly, the standard model does not precisely model compound heterozygotes. These occur when an individual inherits a pair of pathogenic mutations at different sites within the same recessive gene. Typically, the individual would have the disease if the two mutations are out-of-phase (i.e., damaging both gene copies, but not if the mutations are are in-phase (i.e., damaging only one gene copy). Since selection would not act against in-phase compound heterozygotes, the net effect of this is to weaken selection, and *increase* the expected $p_{\text{path}}$ [4, 36]. Since this effect is in the opposite direction of what we see, we do not consider this scenario further.

### Evidence for heterozygous effects.

We next considered an alternative model in which pathogenic variants within recessive disease genes have effects in heterozygotes that are non-clinical, yet large enough to be relevant for selection. Since heterozygotes are so much more common than homozygotes, even weak selection in heterozygotes may be relevant for determining $p_{\text{path}}$ [16].

**Figure 3:**
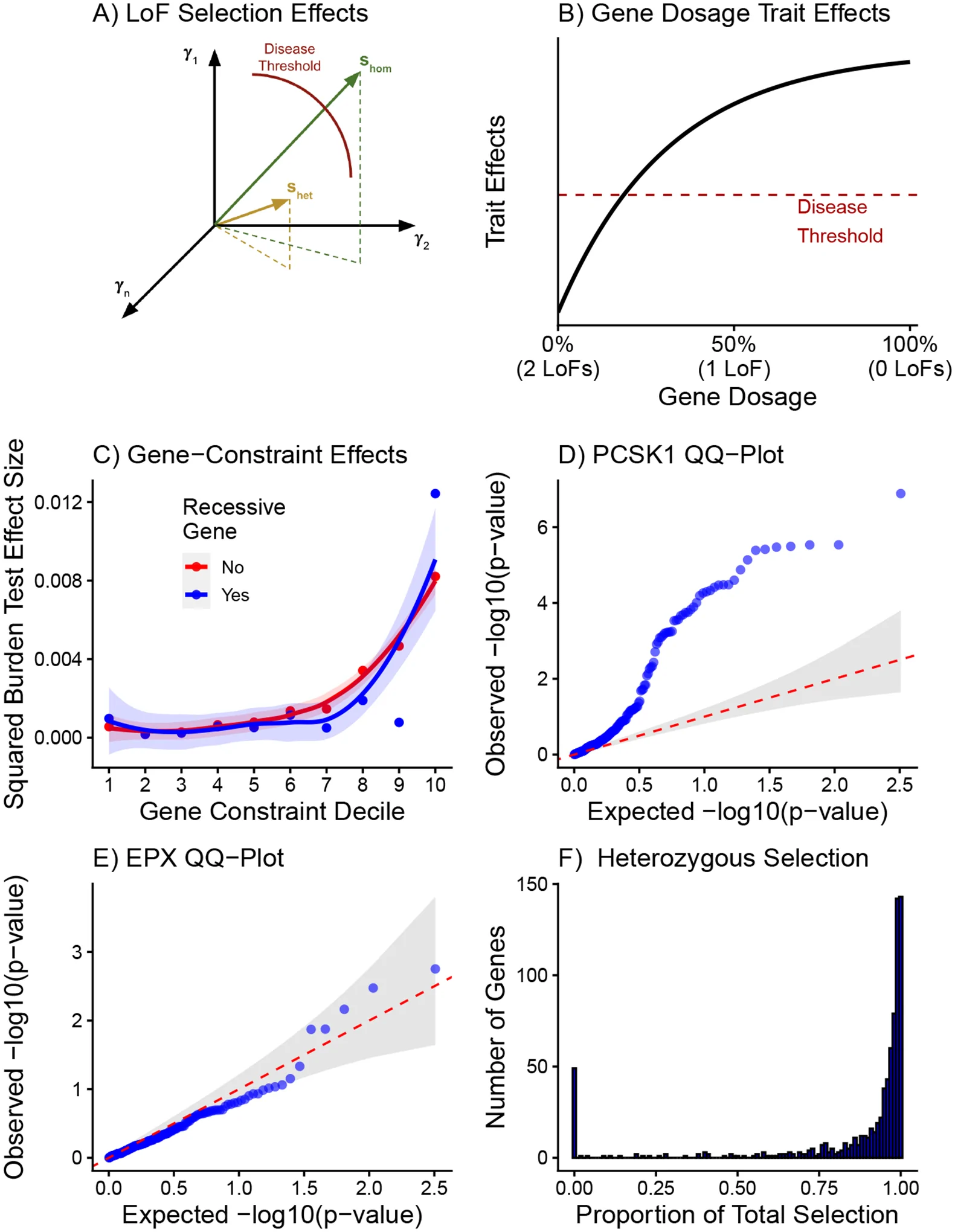
Heterozygous Burden Effects for Recessive Genes: **A)** Variant effects can be conceptualized as vectors in a high-dimensional trait space [37], shown here for hypothetical heterozygous and homozygous LoFs. Here, only the homozygous LoF crosses a clinical threshold for disease, but both genotypes are subject to selection (selection is proportional to the squared magnitude of the vector). **B)** Hypothetical relationship between gene expression dosage and model trait effects [19]. The red line represents the trait effect level where a clinical diagnosis would be made. **C)** Relationship between estimated heterozygous gene constraint decile and average bias-corrected squared LoF burden test effect sizes for 161 quantitative traits, split according to whether or not the genes are classified as recessive. Lines show loess curve fits and standard errors describing the relationship between constraint and squared burden effect sizes. **D)** Quantile-quantile plot of 161 LoF burden test p-values for the recessive PCSK1 gene. **E)** Quantile-quantile plot of 161 LoF burden test p-values for the recessive EPX gene. **F)** Histogram showing the numbers of recessive genes with a given proportion of total selection acting through heterozygous LoFs $\left(\hat{f_{\text{het}}}\right)$.

An emerging body of work shows that, for most genes, the LoF frequency is determined by stabilizing selection against heterozygotes in a high-dimensional space of quantitative traits (Figure 3A). In this model, the selection against LoF heterozygotes $\left(s_{\text{het}}\right)$ is proportional to a sum of squared effect sizes of the LoF on all traits [37–41].

How might this model be relevant to recessive genes? To conceptualize this, we consider the molecular effects of variants that change expression of a relevant gene between 0% (e.g., homozygous LoFs) to 50% (e.g., heterozygous LoFs) to 100% of typical expression. We model these different expression levels as corresponding to different expected values in one of the trait dimensions (Figure 3B) [18–20]. We propose that, for recessive disease genes, a single LoF may impact quantitative traits, while being insufficient to cause a clinical disease state (Figure 3B).

To test this model we sought to measure the effects of heterozygous LoFs at recessive genes on quantitative traits measured by the UK Biobank. We performed LoF burden tests on 19,194 genes, including our curated list of 796 recessive disease genes, for 161 quantitative traits. To ensure that we specifically capture the role of heterozygous LoFs on these quantitative traits, we specifically excluded homozygous LoF individuals from the data analysis.

Our group has previously shown that considering all genes there is a strong relationship between selective constraint and the average squared effect size of LoFs on quantitative traits [41]. When we analyzed the recessive genes in the same way, we find that the heterozygous squared effect sizes are extremely similar to the overall distribution across all genes (Figure 3C).

This result suggests that widespread heterozygous effects on quantitative traits in recessive disease genes in may drive the globally poor fit of the recessive mutationselection balance model.

We also examined the gene×trait significance testing for each recessive gene on each of 161 quantitative traits. Although this test is underpowered due to low LOF frequencies and generally modest effect sizes, we find that 142 of 796 (17.8%) recessive disease genes have significant effects on one or more traits in heterozygotes (Aggregated Cauchy Association Test (ACAT) p-value < 0.05) (Figure S5A). We find qualitatively similar results when we restrict this analysis to 21 uncorrelated quantitative traits (11.8% of traits significant; Figure S5B). These percentages are similar to what we find for the heterozygous effects of all non-recessive genes using the same LoF burden test results (14.6 % for all traits; 14.0 % for uncorrelated traits) (Figure S5C, Figure S5D).

One example of a recessive disease gene with significant heterozygous effects is *PCSK1*, a gene in the same family as *PCSK9* (known for its effects on LDL cholesterol levels [42]). While PCSK1 is known to cause endocrinopathy in an autosomal recessive fashion [43, 44], we find that it has effects on BMI, fat accumulation, and other weight-related phenotypes in heterozygotes (Figure 3D, Figure S5E, Table S3). This indicates that while homozygous LoF of *PCSK1* results in clinical endocrinopathy, variation in PCSK1 expression such as through heterozygous LoFs have effects on quantitative traits, potentially resulting in selection against such variants [45–47].

In contrast, the recessive gene EPX has no observed heterozygous effects and the observed LoF frequency fits recessive mutation-selection balance. *EPX* plays a role in tyrosine nitration granule proteins in resting eosinophils and is recessively linked to eosinophil peroxidase deficiency (Figure 3E, Figure S5F) [48]. While extensive research has identified an association between *EPX* and various phenotypes, such as asthma, allergic response, and airway function, these relationships have primarily been identified in studies with total gene knockouts [48–51]. However, GWAS have identified associations between *EPX* and molecular traits, such as eosinophil and basophil count [52], suggesting that even this gene likely has some degree of phenotypic effects at small-effect variants.

Lastly, we sought to estimate, for each recessive gene, what fraction of selective removal of pathogenic mutations occurs in heterozygotes, which we refer to as $f_{\text{het}}$, and defined:

(5) $$f_{\text{het}}=\frac{2p_{\text{path}}\left(1-p_{\text{path}}\right)s_{\text{het}}}{2p_{\text{path}}\left(1-p_{\text{path}}\right)s_{\text{het}}+2p_{\text{path}}^{2}s_{\text{hom}}}$$

Since $f_{\text{het}}$ is under-determined, we set

(6) $$\hat{f_{het}}=0,\;\text{if}\;p_{\text{path}}\geq \sqrt{\mu L},$$

implying that the allele frequencies are consistent with fully recessive selection; and otherwise we compute a lower bound on $f_{\text{het}}$ as follows:

(7) $$\hat{f_{het}}\leq 1-\frac{p_{\text{path}}^{2}}{\mu L},$$

which makes the conservative assumption that homozygotes have zero fitness, i.e., $s_{\text{hom}}=1$ (Appendix A).

Applying this to empirical data, we find that for most genes, most selection acts through heterozygotes, even when we assume LoFs are homozygous lethal. Out of all recessive genes, only 74 genes (9%) are consistent with primarily being driven by homozygous selection (Figure 3F). In contrast, we estimate that for 73% of annotated recessive genes, at least 90% of the selection occurs in heterozygotes.

### High-frequency recessive genes

**Figure 4:**
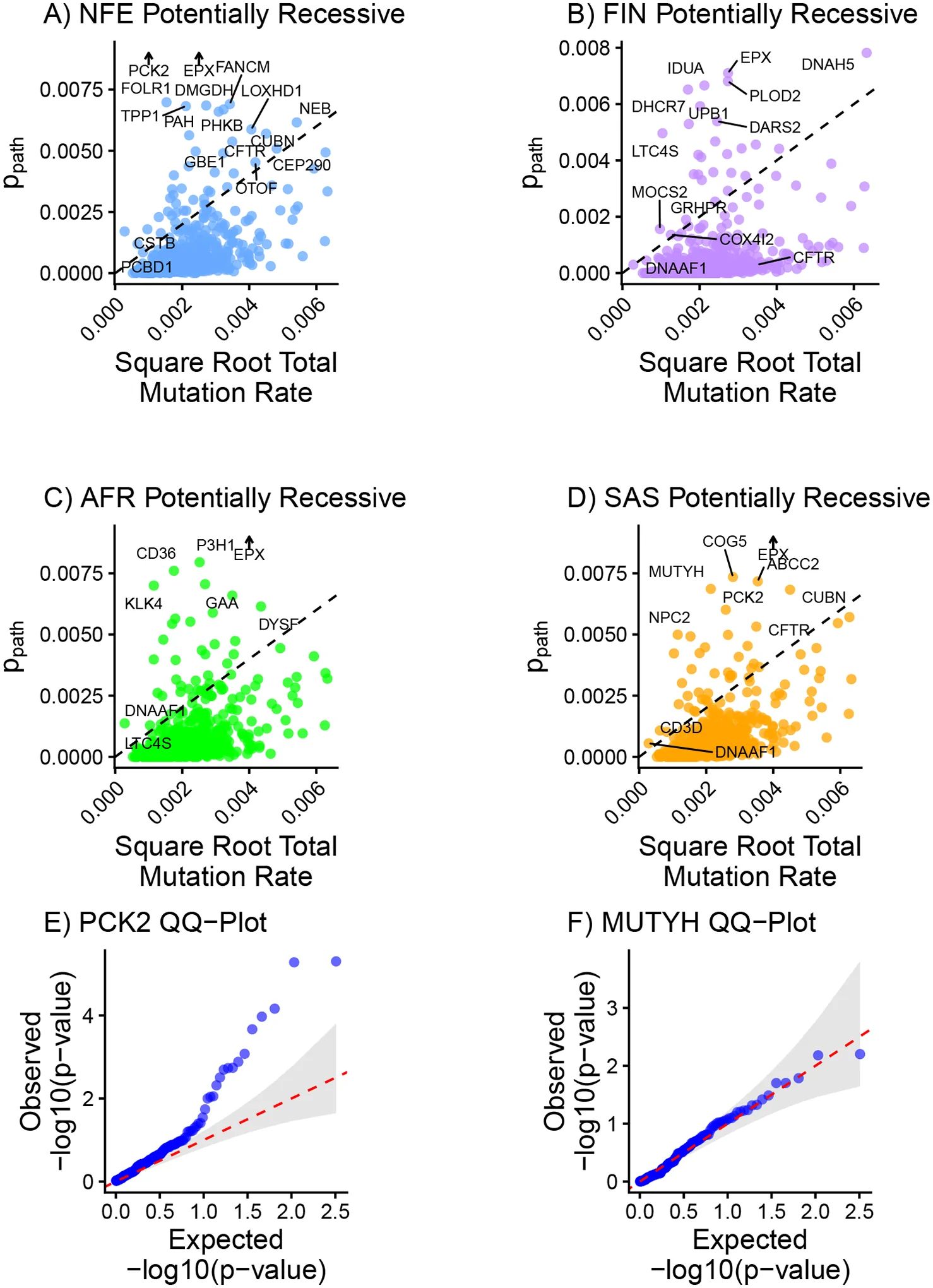
Recessive Genes Under Potentially Recessive Selection: Observed relationship between square root total mutation rate and predicted $p_{\text{path}}$ for recessive disease genes. Chosen recessive disease genes with a predicted $p_{\text{path}}$ in the expected range for recessive mutation-selection balance are labeled using **A)** non-Finnish European (NFE) allele frequencies, **B)** Finnish European (FIN) allele frequencies, **C)** African (AFR) population allele frequencies, and **D)** South-Asian (SAS) population allele frequencies. **E)** Quantilequantile plot of 161 LoF burden test p-values for the recessive PCK2 gene. **F)** Quantile-quantile plot of 161 LoF burden test p-values for the recessive MUTYH gene.

Although most recessive disease genes do not appear to follow recessive mutationselection balance, a handful of genes in each population do have predicted pathogenic variation frequencies within the range consistent with the fully-recessive model (Figure 4A). While the specific sets of high-frequency genes vary (Figure 4B, Figure 4C, Figure 4D, Table S4), the absolute numbers are quite similar across populations (**49 in NFE, 34 in FIN, 39 in AFR, and 43 in SAS**).

Genetic drift, and potential variant ascertainment bias [26] may explain the differences between populations, and this stochasticity may result in some genes having higher than expected $p_{\text{path}}$. Alternatively, the difference in which genes are consistent with no selection against heterozygotes may be driven by different selective pressures in different populations, for example due to differing environments. Finally, here we only considered selection *against* LoFs, but these genes with high $p_{\text{path}}$ could also be driven by balancing selection or positive selection [53, 54].

When we examined in different populations genes previously believed to affected by balancing selection [53, 55–58], we observed variation in the genes that fit recessive mutation-selection balance (Figure S6A, Figure S6B, Figure S6C, Figure S6D). For example, two recessive disease genes that fit mutation-selection balance based on the study population and are believed to be affected by balancing selection are *PAH* (Figure S6A) and *CFTR* (Figure S6D), which are the genes associated with causing phenylketonuria (PKU) [59] and cystic fibrosis [56], respectively. If these genes and other recessive disease genes are under the influence of balancing selection, their $p_{\text{path}}$ would be higher than expected under a model of purely recessive selection, and potentially fit recessive mutation-selection balance based on the specific population analyzed, due to differences in selective pressures, and choice of variant or LoF inclusion criteria.

Alternatively, this analysis may identify genes where the selection pressures acting on the gene and affecting $p_{\text{path}}$ are unknown. A potential example among these recessive genes is *PCK2*, a mitochondrial isozyme of *PEPCK*, which has a well-known function in gluconeogenesis [60]. PCK2 is consistent with having no selection against heterozygotes in both the NFE and SAS populations (Figure 4A, Figure 4D), and possesses particularly high $p_{\text{path}}$ in the NFE population (Figure 4A, Table S4). When examining the phenotypic effects of this gene, we observe a wide range of heterozygous effects on the 161 traits we analyzed (Figure 4E), and other research has found that over-expression of *PCK2* is linked to lung cancer risk, diabetes, and obesity [61, 62]. This suggests that perhaps any selection against *PCK2* in heterozygotes caused by these phenotypic consequences is offset by some other, unknown fitness advantage.

All together, these results indicate that even these genes that are consistent with purely recessive selection may have effects in heterozygotes, and need not follow the textbook definition of recessive selection.

On the other hand, some genes appear to be purely recessive by all measures we examined. For example, *MUTYH* fits recessive mutation-selection balance (Figure 4D) and has no observed heterozygous effects (Figure 4F). *MUTYH* has a role in base excision repair and is recessively associated with colorectal adenoma risk [63]. Recent research has found that *MUTYH* continues to function in suppressing mutations even in the heterozygous LoF state [64], and only when two LoFs are present does the rate of *de novo* mutations increase, resulting in increased cancer risk. Overall, it seems plausible that *MUTYH* is an example of a gene governed by recessive mutation-selection balance.

We also examined the subset of recessive genes that match the predictions of purely recessive mutation-selection balance in the NFE, FIN, AFR, and SAS populations (Figure 4A, Figure 4B, Figure 4C, Figure 4D) to identify whether these genes showed any particular patterns. The associated diseases as well as cellular functions of these genes were varied and not limited to a specific biological function or system. However, genes that fit mutation-selection balance in the Non-Finnish European population are enriched compared to all recessive disease genes for polarized epithelial cellular component GO terms (Table S5, p-value < 0.05), which are involved in transport and environmental sensing and have associated with a variety of diseases including immunological effects [65–67], possibly indicating positive or balancing selection driven by host/pathogen competition [68–71].

## Discussion

The role of heterozygous and homozygous selection on the equilibrium allele frequency of pathogenic variation in a gene has been understood theoretically for nearly a century [2]. Purely recessive mutation-selection balance is a classic result found in textbooks and often taught in introductory population genetics courses [72, 73]. Meanwhile, the study of Mendelian autosomal recessive genes might lead one to believe that many genes should be governed by purely recessive mutation-selection balance.

Here, we find purely recessive mutation-selection balance is a poor fit for nearly every protein coding gene in the human genome, including recessive disease genes. Our analyses reveal that inbreeding is an unlikely explanation for this phenomenon, and observed $p_{\text{LoF}}$ are instead likely shaped by selection against heterozygotes. We find that heterozygous LoFs in recessive disease genes have quantitative effects that are similar to genes overall. While these effects might not rise to the level of being classified as Mendelian disorders, the substantially larger frequency of individuals with heterozygous LoFs causes this selection against heterozygotes to be the primary determinant of $p_{\text{LoF}}$ even in recessive disease genes.

Our results suggest that recessive disease genes are often highly pleiotropic, with effects even in heterozygotes, and hence could play roles in many complex diseases. As a result, any model of selection in humans should default to assuming that selection occurring in heterozygotes is a major factor.

Another aspect of our study worth considering is that the traits tested for heterozygous effects through burden tests are relatively limited. While these traits were chosen to represent a range of continuous non-disease, biochemistry, and anthropometric traits, they are far from exhaustive. Thus, our results provide a conservative estimate of the role of heterozygous pathogenic variants in recessive disease genes, and inclusion of more uncorrelated traits, particularly molecular traits, should only reveal an even broader range of heterozygous effects of variants in recessive genes [74–76].

Furthermore, it is difficult to determine which recessive genes with unaccounted for heterozygous effects and selection are classified as recessive because these genes are understudied or if their heterozygous effects are too small to be clinically relevant. For example, recessive genes such as *PCSK1* have strong heterozygous effects that are easily identified through LoF burden tests. While identifying these effects can inform our understanding of a wide range of quantitative traits, classifying these strong effect genes as recessive may simply be due to a lack of research. If these genes were excluded, the mutation-selection balance model may appear more valid in its current form. Inversely, there are a large number of recessive genes where we infer that the majority of selection acts through heterozygotes, but no significant gene-trait effects were identified. This can be partially attributed to the previously mentioned issue of a lack of tested traits or a lack of power, insofar as current datasets may be too small to identify individual heterozygous effects that are captured by measurements of selection, which account for the effect of variations in these genes over many generations. Identifying this second class of genes with known recessive effects and minuscule additive effects is particularly crucial for understanding human genetic variation.

In summary, we have explored the relationship between equilibrium allele frequency and natural selection on complex traits. Our results highlight the potential for widespread effects of heterozygous LoFs, even in recessive disease genes. These insights about recessive genes and natural selection will be informative for various downstream analyses, including the study of the genetic architecture of complex traits and investigations of the selective pressures underlying human genetic variation.

## Methods

### Identifying Recessive Genes

An initial list of 1,183 recessive genes was curated by previous studies [16, 22, 23] by inclusion in Online Mendelian Inheritance in Man (OMIM), a comprehensive compendium of human genes and genetic phenotypes [24], and manual curation through other resources such PubMed and Gene Reviews [77, 78]. To increase the likelihood that the list of recessive genes only affected traits through autosomal recessive inheritance, the initial list of recessive genes was cross-referenced against the OMIM database in December 2024, and any genes with non-autosomal recessive inheritance were excluded in order to produce the most updated list of 796 genes associated with recessive traits.

### Gene Data

Gene-level information about loss-of-function (LoF) variants, including allele frequencies and mutation locations, were retrieved from the gnomAD database [5]. For this study, we used gnomAD v4.0 genomic information from 705,148 unrelated individuals in the Non-Finnish European, Finnish, African, and South Asian ancestry groups. We defined LoFs as those predicted to cause stop-gain, slice-acceptor, and splice-donor effects using the Variant Effect Predictor (VEP; v85) [79]. When calculating gene-level LoF allele frequencies $\left(p_{\text{LoF}}\right)$, we summed allele frequencies for LoFs within the gene, excluding variants with an allele frequency > 0.005 to exclude LoFs with potential bioinformatic errors [10, 26]. (Our overall conclusions are robust to excluding this filter.) For each gene, $p_{\text{LoF}}$ was calculated separately for each population. This variant inclusion/exclusion criteria potentially excludes many pathogenic missense and LoF variants that may lead to a recessive disease in these genes [80]. However, the chosen variants are known to be highly likely to stop gene function and increases our confidence in examining pathogenic variation and its effect in recessive mutation-selection balance [5, 25, 26]. Total mutation rates for each gene were calculated by considering all possible LoF-causing point mutations and using a mutation rate that accounts for trinucleotide context and methylation status at CpGs as described in [5, 25]. Final population-specific $p_{\text{LoF}}\text{s}$ and total mutation rates along with the previously-determined recessive gene status were used to visualize additive and recessive mutation-selection balance.

### Adjusting Mutation-Selection Balance for Missing Variation

We wanted to estimate the proportion of pathogenic variants that might be excluded from the types of variants we considered above: stop-gain, splice-donor-disrupting, and splice-acceptor-disrupting point mutations. In particular, we identified the percentage of pathogenic variation in 25 well-known recessive disease genes represented by these classed of variants [81, 82]. Using ClinVar [83] to identify pathogenic and likely pathogenic short variation in recessive disease genes we analyzed genes including *HBB, CFTR, PAH, ATP7B, MUTYH, HEXA, GBA1, USH2A*, and *SMN1*, which are the genes associated with sickle cell anemia, cystic fibrosis, phenylketonuria (PKU), Wilson disease, MUTYH-associated gastric cancer, Tay-sachs disease, Gaucher disease, Usher syndrome, and spinal muscular atrophy, respectively [13, 14, 55, 63, 84–88]. After using the ClinVar-provided molecular consequences to identify LoFs and non-LoFs, we calculated the percentage of unique pathogenic variation that were predicted to cause stop gain, disrupt a splice donor, or disrupt a splice acceptor. For each gene, the inverse of these percentages was calculated as a conversion factor to adjust for potential missing non-LoF pathogenic variation, and we adjusted the visualizations of recessive mutation-selection balance by increasing $p_{\text{LoF}}$ by the average of the calculated conversion factors and the square root total mutation rates by the square root of the average of the conversion factors.

To determine the conversion factors required so that 50% and 90% of recessive disease genes fit the recessive mutation-selection balance model, we identified the recessive disease gene whose ratio between the LoF allele frequency, $p_{\text{LOF}}$, and square root total mutation rate, $\sqrt{\mu L}$, is at the 50^*th*^ and 10^*th*^ percentile of all recessive disease gene ratios, respectively. From the ratios of the genes at the 50^*th*^ and 10^*th*^ percentile, the conversion factors are the square of the inverse of the ratio for the gene (Equation 8).


(8) $$\text{Conversion-Factor}={\left(\frac{1}{\text{ratio}}\right)}^{2}={\left(\frac{1}{\frac{p_{\text{LoF}}}{\sqrt{\mu L}}}\right)}^{2}={\left(\frac{\sqrt{\mu L}}{p_{\text{LoF}}}\right)}^{2}$$

### Mutation-Selection Balance with Inbreeding

Along with the classic mutation-selection balance model (Equation 4), we adjusted another model by Nei that calculates the expected equilibrium $p_{\text{LoF}}$ in a finite population (Appendix A) [31] in order to account for the effect of inbreeding depression on recessive genes in a more realistic population (Equation 9).


(9) $$E[p_{\text{path}}]=\frac{\sum_{k=0}^{\infty }\frac{\left(-4Ns_{\text{hom}}\varphi /(1+\varphi {)}^{k}\Gamma (2N\mu /(1+\varphi )+(k+1)/2)\right.}{k!{\left(2Ns_{\text{hom}}(1-\varphi )/(1+\varphi )\right)}^{2N\mu /(1+\varphi )+(k+1)/2}}}{\sum_{\ell =0}^{\infty }\frac{\left(-4Ns_{\text{hom}}\varphi /(1+\varphi {)}^{\ell }\Gamma (2N\mu /(1+\varphi )+\ell /2)\right.}{\ell !{\left(2Ns_{\text{hom}}(1-\varphi )/(1+\varphi )\right)}^{2N\mu /(1+\varphi )+\ell /2}}}$$

With these two models, we can predict $p_{\text{LoF}}$ under recessive lethal selection for different total mutation rates and inbreeding values, providing a lower bound for values of $p_{\text{LoF}}$ consistent with a purely recessive model.

### Simulating Populations with Varied Inbreeding Coefficients

In order to assess the fit of Equations 1 and 3 for varying levels of inbreeding assuming recessive lethality, we used SLiM v4.3 to generate simulated population data [89]. All simulated populations contained 20,000 individuals and possessed a single recessive lethal gene that accumulated LoF variants over 200,000 generations. We generated 125 simulated populations each with 25 varying and evenly spaced total mutation rates ([0–0.005]) and inbreeding coefficients ([0, 1/64, 1/32, 1/16, 1/8]). After simulating and calculating $p_{\text{LoF}}$ for each population, we generated theoretical model values for the same parameters using Equation 4 and Equation 9.

The simulated data were plotted over the predicted lower bounds for LoF allele frequency under different levels of inbreeding (Equation 4 and Equation 9) to assess fit. Additionally, empirical recessive gene data was compared to both models under varying inbreeding conditions to determine the required level of inbreeding for recessive genes to be consistent with purely recessive mutation-selection balance.

### Burden Tests

We used REGENIE [90] to perform gene-level burden tests on 161 quantitative traits available in UK BioBank (UKB) [91]. For the whole-genome regression, which is the first step in REGENIE, SNPs from the genotyping array were pruned with a 1000 variant sliding window with 100 variant shifts and an $R^{2}$ threshold of 0.9. SNPs were then filtered to have MAF > 1%, genotype missingness < 10%, and Hardy-Weinberg equilibrium (HWE) test p-value > 10^−15^. For LoF burden tests, we used the following covariates: age, sex, age-by-sex, age squared, 15 genotyping PCs, 20 rare variant PCs, and WES batch. After whole-genome regression, we used the second step of REGENIE to perform burden tests using the whole-exome sequencing data. We used the LOFTEE plugin in Ensembl's Variant Effect Predictor to annotate high-confidence LoF sites [5, 79]. We further filtered any annotated LoF site with MAF < 1% using a variablefrequency filter across genes based on their constraint, as described previously [10, 20]. For each gene, we filtered to individuals that carried at most one LoF variant, ensuring that burden tests were performed using only LoF heterozygotes. Phenotypes were rank-inverse-normal transformed in both the first and second step. The same covariates were used in both steps.

To generate a list of genetically uncorrelated traits, we used previously released estimates of genetic correlation based on common variants from the UKB (http://www.nealelab.is/uk-biobank/). Any traits with genetic correlation greater than 0.75 were clustered together, and traits with the most signal based on biascorrected average squared burden effect size were picked from the clusters to generate a list of 21 uncorrelated traits.

### Measuring Burden Effects Across Gene Constraint

The estimated heterozygous gene constraint was calculated for all genes to determine if there was a positive relationship between heterozygous selection and heterozygous trait effects. For all genes, including recessive genes, the heterozygous gene constraint was estimated by dividing the gene's total mutation rate by $p_{\text{Lof}}$ as modeled by additive mutation-selection balance (Figure 1A). In addition, in order to account for statistical estimation bias, we calculated the bias-corrected squared effect size for all gene-trait LoF burden tests by subtracting the squared standard error of the test from the squared estimated effect size. Bias-corrected squared effect sizes were then averaged by gene to provide all genes an estimated gene constraint and average squared burden test effect size. All genes were then segregated into deciles based on estimated gene constraint. Average squared burden test effect sizes for each gene were averaged again by bin and recessive gene status. Final deciles and decile effect sizes were lastly separated by recessive gene status and plotted to visualize the relationship between constraint and heterozygous effect size.

### Calculating Proportionate Heterozygous Selection

In order to estimate a lower bound of the effect of heterozygous selection on recessive genes, we simplified the mutation-selection balance model $(2\mu L=2p_{\text{path}}(1-\left.\left.p_{\text{path}}\right)s_{\text{het}}+2p_{\text{path}}^{2}s_{\text{hom}}\right)$ to compute $s_{\text{het}}$ given the gene's $p_{\text{path}}$ and total mutation rate, and assuming the recessive gene was recessive lethal, $s_{\text{hom}}=1$. Details on the estimator are provided in Appendix A. We then used this estimate to calculate the proportion of heterozygous selection versus total selection (the combination of heterozygous and homozygous selection), for each gene with the same assumption of recessive lethality. This provides an analytical lower bound for the proportion of total selection acting on each gene through heterozygotes.

### Recessive Gene QQ Plots for Quantitative Traits

Quantile-quantile plots were generated for all recessive genes using -log10(p-values) generated from LoF burden tests for a list of 161 UKB quantitative traits and subset of 21 uncorrelated quantitative traits. Observed -log10(p-values) were compared to expected values from a uniform distribution. 95% confidence intervals were generated from beta distribution using the 2.5% and 97.5% for the i-th smallest expected p-value of the uniform distribution.

### Aggregated Cauchy Association Tests

For each recessive disease gene, Aggregated Cauchy Association Test (ACAT) was used to combine all LoF burden test p-values for the gene [92]. We performed this analysis on different sets of burden test results: 1) combining LoF burden tests for all 161 UKB quantitative traits, and 2) combining LoF burden tests for 21 uncorrelated quantitative traits. This analysis would identify recessive genes with significant non-recessive effects using the available trait data. This analysis was repeated for all 18,398 non-recessive genes independent of the our curated recessive disease gene list. With all the non-recessive genes, ACAT was used to combine all LoF burden test p-values for each gene across the LoF burden tests for all 161 UKB quantitative traits and LoF burden tests for 21 uncorrelated quantitative traits.

### Identifying and Analyzing Purely Recessive Genes

Recessive genes that possessed a $p_{\text{LoF}}$ greater than their respective square root total mutation rate were identified to follow classic recessive mutation-selection balance and potentially only be affected by homozygous selection. As population-specific $p_{\text{LoF}}$ were calculated, the list of “purely” recessive genes varied by population and were individually identified.

Gene Ontology (GO) enrichment analysis was performed on recessive genes identified as “purely” recessive in the non-Finnish European, Finnish, African, and South Asian populations in order to identify shared biological, molecular, or cellular function among identified recessive genes [93, 94].

### Mutation-Selection Balance with Balancing Selection

In order to understand the potential role of balancing selection on the mutationselection balance for genes associated with recessive diseases, we visualized the relationship between $p_{\text{LoF}}$ and square root total mutation rate for genes previously suspected to be affected by balancing selection and are associated with only recessive diseases [53, 55–58]. We performed this analysis using observed $p_{\text{LoF}}$ from NFE, FIN, AFR, and SAS populations available from gnomAD v4.0 genomic information.

## Supplementary Material

Supplement 1

1

## References

[1] Haldane J. B. S.. “A Mathematical Theory of Natural and Artificial Selection, Part V: Selection and Mutation”. en. In: Mathematical Proceedings of the Cambridge Philosophical Society 23.7 (July 1927), pp. 838–844. ISSN: 1469-8064, 0305-0041. DOI: 10.1017/S0305004100015644.

[2] Haldane J. B. S.. “The Effect of Variation of Fitness”. In: The American Naturalist 71.735 (July 1937), pp. 337–349. ISSN: 0003-0147. DOI: 10.1086/280722.

[3] Lek Monkol “Analysis of protein-coding genetic variation in 60,706 humans”. en. In: Nature 536.7616 (Aug. 2016). Publisher: Nature Publishing Group, pp. 285–291. ISSN: 1476-4687. DOI: 10.1038/nature19057. PMC5018207 27535533

[4] Guo Michael H. “Inferring compound heterozygosity from largescale exome sequencing data”. en. In: Nature Genetics 56.1 (Jan. 2024). Publisher: Nature Publishing Group, pp. 152–161. ISSN: 1546-1718. DOI: 10.1038/s41588-023-01608-3. PMC10872287 38057443

[5] Karczewski Konrad J. “The Mutational Constraint Spectrum Quantified from Variation in 141,456 Humans”. In: Nature 581.7809 (May 2020), pp. 434–443. ISSN: 1476-4687. DOI: 10.1038/s41586-020-2308-7. PMC7334197 32461654

[6] Chen Siwei “A Genomic Mutational Constraint Map Using Variation in 76,156 Human Genomes”. In: Nature 625.7993 (Jan. 2024), pp. 92–100. ISSN: 1476-4687. DOI: 10.1038/s41586-023-06045-0. PMC11629659 38057664

[7] Cassa Christopher A. “Estimating the selective effects of heterozygous protein-truncating variants from human exome data”. en. In: Nature Genetics 49.5 (May 2017). Publisher: Nature Publishing Group, pp. 806–810. ISSN: 1546-1718. DOI: 10.1038/ng.3831. PMC5618255 28369035

[8] Fuller Zachary L. “Measuring Intolerance to Mutation in Human Genetics”. In: Nature Genetics 51.5 (May 2019), pp. 772–776. ISSN: 1546-1718. DOI: 10.1038/s41588-019-0383-1. PMC6615471 30962618

[9] Agarwal Ipsita “Relating pathogenic loss-of-function mutations in humans to their evolutionary fitness costs”. In: eLife 12 (Jan. 2023). Ed. by Perry George H. Publisher: eLife Sciences Publications, Ltd, e83172. ISSN: 2050-084X. DOI: 10.7554/eLife.83172. PMC9937649 36648429

[10] Zeng Tony “Bayesian Estimation of Gene Constraint from an Evolutionary Model with Gene Features”. In: Nature Genetics (July 8, 2024), pp. 1–12. ISSN: 1546-1718. DOI: 10.1038/s41588-024-01820-9. 38977852

[11] Ong Thida and Ramsey Bonnie W.. “Cystic Fibrosis: A Review”. In: JAMA 329.21 (June 6, 2023), pp. 1859–1871. ISSN: 0098-7484. DOI: 10.1001/jama.2023.8120. 37278811

[12] McKusick Victor A.. Mendelian Inheritance in Man: Catalogs of Autosomal Dominant, Autosomal Recessive, and X-Linked Phenotypes. Elsevier, May 12, 2014.368 pp. ISBN: 978-1-4831-9427-1. Google Books: xzGRBQAAQBAJ.

[13] Filho Jose Americo Fernandes and Shapiro Barbara E.. “Tay-Sachs Disease”. In: Archives of Neurology 61.9 (Sept. 1, 2004), pp. 1466–1468. ISSN: 0003-9942. DOI: 10.1001/archneur.61.9.1466. 15364698

[14] Lonergan Gael J., Cline David B., and Abbondanzo Susan L.. “Sickle Cell Anemia”. In: RadioGraphics 21.4 (July 2001), pp. 971–994. ISSN: 0271-5333. DOI: 10.1148/radiographics.21.4.g01j123971. 11452073

[15] Balick Daniel J. “Overcoming Constraints on the Detection of Recessive Selection in Human Genes from Population Frequency Data”. In: The American Journal of Human Genetics 109.1 (Jan. 6, 2022), pp. 33–49. ISSN: 0002-9297, 1537-6605. DOI: 10.1016/j.ajhg.2021.12.001. PMC8764206 34951958

[16] Amorim Carlos Eduardo G. “The Population Genetics of Human Disease: The Case of Recessive, Lethal Mutations”. In: PLOS Genetics 13.9 (Sept. 28, 2017), e1006915. ISSN: 1553-7404. DOI: 10.1371/journal.pgen.1006915. PMC5619689 28957316

[17] Boyle Evan A., Li Yang I., and Pritchard Jonathan K.. “An Expanded View of Complex Traits: From Polygenic to Omnigenic”. In: Cell 169.7 (June 15, 2017), pp. 1177–1186. ISSN: 0092-8674, 1097-4172. DOI: 10.1016/j.cell.2017.05.038. PMC5536862 28622505

[18] Domingo Júlia Nonlinear transcriptional responses to gradual modulation of transcription factor dosage. en. ISSN: 2692-8205 Pages: 2024.03.01.582837 Section: New Results. Aug. 2025. DOI: 10.1101/2024.03.01.582837. PMC12803512 41532405

[19] Naqvi Sahin “Precise modulation of transcription factor levels identifies features underlying dosage sensitivity”. en. In: Nature Genetics 55.5 (May 2023). Publisher: Nature Publishing Group, pp. 841–851. ISSN: 1546-1718. DOI: 10.1038/s41588-023-01366-2. PMC10181932 37024583

[20] Milind Nikhil Buffering and Non-Monotonic Behavior of Gene Dosage Response Curves for Human Complex Traits. Nov. 11, 2024. DOI: 10.1101/2024.11.11.24317065. URL: https://www.medrxiv.org/content/10.1101/2024.11.11.24317065v1 (visited on 12/05/2024). Pre-published.

[21] Seal Ruth L “Genenames.org: the HGNC resources in 2023”. In: Nucleic Acids Research 51.D1 (Jan. 2023), pp. D1003–D1009. ISSN: 0305-1048. DOI: 10.1093/nar/gkac888. PMC9825485 36243972

[22] Berg Jonathan S. “An Informatics Approach to Analyzing the Incidentalome”. In: Genetics in Medicine 15.1 (Jan. 1, 2013), pp. 36–44. ISSN: 1098-3600, 1530-0366. DOI: 10.1038/gim.2012.112. PMC3538953 22995991

[23] Blekhman Ran “Natural Selection on Genes That Underlie Human Disease Susceptibility”. In: Current Biology 18.12 (June 24, 2008), pp. 883–889. ISSN: 0960-9822. DOI: 10.1016/j.cub.2008.04.074. PMC2474766 18571414

[24] Amberger Joanna S. “OMIM.Org: Online Mendelian Inheritance in Man (OMIM^®^), an Online Catalog of Human Genes and Genetic Disorders”. In: Nucleic Acids Research 43.D1 (Jan. 28, 2015), pp. D789–D798. ISSN: 0305-1048. DOI: 10.1093/nar/gku1205. PMC4383985 25428349

[25] Cummings Beryl B. “Transcript expression-aware annotation improves rare variant interpretation”. en. In: Nature 581.7809 (May 2020). Publisher: Nature Publishing Group, pp. 452–458. ISSN: 1476-4687. DOI: 10.1038/s41586-020-2329-2. PMC7334198 32461655

[26] Stolyarova Anastasia, Coop Graham, and Przeworski Molly. “The Distribution of Highly Deleterious Variants across Human Ancestry Groups”. In: Proceedings of the National Academy of Sciences 122.21 (May 27, 2025), e2503857122. DOI: 10.1073/pnas.2503857122. PMC12130832 40408403

[27] Charlesworth Deborah and Willis John H.. “The Genetics of Inbreeding Depression”. In: Nature Reviews Genetics 10.11 (Nov. 2009), pp. 783–796. ISSN: 1471-0064. DOI: 10.1038/nrg2664. 19834483

[28] Durbin Richard M. “A map of human genome variation from population-scale sequencing”. en. In: Nature 467.7319 (Oct. 2010). Publisher: Nature Publishing Group, pp. 1061–1073. ISSN: 1476-4687. DOI: 10.1038/nature09534. PMC3042601 20981092

[29] Gazal Steven “High Level of Inbreeding in Final Phase of 1000 Genomes Project”. In: Scientific Reports 5.1 (Dec. 2, 2015), p. 17453. ISSN: 2045-2322. DOI: 10.1038/srep17453. PMC4667178 26625947

[30] Yengo Loic, Wray Naomi R., and Visscher Peter M.. “Extreme inbreeding in a European ancestry sample from the contemporary UK population”. en. In: Nature Communications 10.1 (Sept. 2019). Publisher: Nature Publishing Group, p. 3719. ISSN: 2041-1723. DOI: 10.1038/s41467-019-11724-6. PMC6722066 31481654

[31] Nei M. “The Frequency Distribution of Lethal Chromosomes in Finite Populations.” In: Proceedings of the National Academy of Sciences 60.2 (June 1968), pp. 517–524. DOI: 10.1073/pnas.60.2.517. PMC225078 5248809

[32] Laws R. J. and Jamieson I. G.. “Is lack of evidence of inbreeding depression in a threatened New Zealand robin indicative of reduced genetic load?” en. In: Animal Conservation 14.1 (2011)._eprint: https://zslpublications.onlinelibrary.wiley.1795.2010.00388.x, pp. 47–55. ISSN: 1469-1795. DOI: 10.1111/j.1469-1795.2010.00388.x.

[33] Hedrick Philip W. and Garcia-Dorado Aurora. “Understanding Inbreeding Depression, Purging, and Genetic Rescue”. English. In: Trends in Ecology & Evolution 31.12 (Dec. 2016). Publisher: Elsevier, pp. 940–952. ISSN: 0169-5347. DOI: 10.1016/j.tree.2016.09.005. 27743611

[34] Robinson Jacqueline A. “Purging of Strongly Deleterious Mutations Explains Long-Term Persistence and Absence of Inbreeding Depression in Island Foxes”. English. In: Current Biology 28.21 (Nov. 2018). Publisher: Elsevier, 3487-3494.e4. ISSN: 0960-9822. DOI: 10.1016/j.cub.2018.08.066. PMC6462144 30415705

[35] Wall Jeffrey D. “South Asian medical cohorts reveal strong founder effects and high rates of homozygosity”. en. In: Nature Communications 14.1 (June 2023). Publisher: Nature Publishing Group, p. 3377. ISSN: 2041-1723. DOI: 10.1038/s41467-023-38766-1. PMC10250394 37291107

[36] Lassen Frederik H. “Exome-wide evidence of compound heterozygous effects across common phenotypes in the UK Biobank”. English. In: Cell Genomics 4.7 (July 2024). Publisher: Elsevier. ISSN: 2666-979X. DOI: 10.1016/j.xgen.2024.100602. PMC11293579 38944039

[37] Simons Yuval B. “A Population Genetic Interpretation of GWAS Findings for Human Quantitative Traits”. In: PLOS Biology 16.3 (Mar. 16, 2018), e2002985. ISSN: 1545-7885. DOI: 10.1371/journal.pbio.2002985. PMC5871013 29547617

[38] Sanjak Jaleal S. “Evidence of Directional and Stabilizing Selection in Contemporary Humans”. In: Proceedings of the National Academy of Sciences 115.1 (Jan. 2, 2018), pp. 151–156. DOI: 10.1073/pnas.1707227114. PMC5776788 29255044

[39] Sella Guy and Barton Nicholas H.. “Thinking About the Evolution of Complex Traits in the Era of Genome-Wide Association Studies”. en. In: Annual Review of Genomics and Human Genetics 20.1 (Aug. 2019), pp. 461–493. ISSN: 1527-8204, 1545-293X. DOI: 10.1146/annurev-genom-083115-022316. 31283361

[40] Simons Yuval B. Simple Scaling Laws Control the Genetic Architectures of Human Complex Traits. Oct. 7, 2022. DOI: 10.1101/2022.10.04.509926. URL: https://www.biorxiv.org/content/10.1101/2022.10.04.509926v1 (visited on 07/29/2025). Pre-published. PMC12517483 41082512

[41] Spence Jeffrey P. “Specificity, length and luck drive gene rankings in association studies”. en. In: Nature (Nov. 2025). Publisher: Nature Publishing Group, pp. 1–8. ISSN: 1476-4687. DOI: 10.1038/s41586-025-09703-7. PMC12823407 41193809

[42] Cariou Bertrand, May Cédric Le, and Costet Philippe. “Clinical Aspects of PCSK9”. In: Atherosclerosis 216.2 (June 1, 2011), pp. 258–265. ISSN:0021-9150. DOI: 10.1016/j.atherosclerosis.2011.04.018. 21596380

[43] Jackson Robert S. “Small-intestinal dysfunction accompanies the complex endocrinopathy of human proprotein convertase 1 deficiency”. en. In: The Journal of Clinical Investigation 112.10 (Nov. 2003). Publisher: American Society for Clinical Investigation, pp. 1550–1560. ISSN: 0021-9738. DOI: 10.1172/JCI18784. PMC259128 14617756

[44] Cool David R “Carboxypeptidase E Is a Regulated Secretory Pathway Sorting Receptor: Genetic Obliteration Leads to Endocrine Disorders in Cpefat Mice”. In: Cell 88.1 (Jan. 1997), pp. 73–83. ISSN: 0092-8674. DOI: 10.1016/S0092-8674(00)81860-7. 9019408

[45] Ramos-Molina B., Martin M. G., and Lindberg I.. “PCSK1 Variants and Human Obesity”. In: Progress in Molecular Biology and Translational Science. Ed. by Tao Ya-Xiong. Vol. 140. Genetics of Monogenic and Syndromic Obesity. Academic Press, Jan. 1, 2016, pp. 47–74. DOI: 10.1016/bs.pmbts.2015.12.001. PMC6082390 27288825

[46] Stijnen Pieter “The Association of Common Variants in PCSK1 With Obesity: A HuGE Review and Meta-Analysis”. In: American Journal of Epidemiology 180.11 (Dec. 1, 2014), pp. 1051–1065. ISSN: 0002-9262. DOI: 10.1093/aje/kwu237. 25355447

[47] Stijnen Pieter “PCSK1 Mutations and Human Endocrinopathies: From Obesity to Gastrointestinal Disorders”. In: Endocrine Reviews 37.4 (Aug. 1, 2016), pp. 347–371. ISSN: 0163-769X. DOI: 10.1210/er.2015-1117. 27187081

[48] Percopo Caroline M “Impact of Eosinophil-Peroxidase (EPX) Deficiency on Eosinophil Structure and Function in Mouse Airways”. In: Journal of Leukocyte Biology 105.1 (Jan. 1, 2019), pp. 151–161. ISSN: 0741-5400. DOI: 10.1002/JLB.3AB0318-090RR. PMC6310085 30285291

[49] Klonoff-Cohen Hillary and Polavarapu Mounika. “Eosinophil Protein X and Childhood Asthma: A Systematic Review and Meta-Analysis”. In: Immunity, Inflammation and Disease 4.2 (2016), pp. 114–134. ISSN: 2050-4527. DOI: 10.1002/iid3.104. PMC4879459 27957324

[50] Nair P. “Eosinophil Peroxidase in Sputum Represents a Unique Biomarker of Airway Eosinophilia”. In: Allergy 68.9 (2013), pp. 1177–1184. ISSN: 1398-9995. DOI: 10.1111/all.12206. PMC3788081 23931643

[51] Ravi Acharya K. and Ackerman Steven J.. “Eosinophil Granule Proteins: Form and Function”. In: Journal of Biological Chemistry 289.25 (June 1, 2014), pp. 17406–17415. ISSN: 0021-9258. DOI: 10.1074/jbc.R113.546218. PMC4067173 24802755

[52] Barton Alison R. “Whole-exome imputation within UK Biobank powers rare coding variant association and fine-mapping analyses”. en. In: Nature Genetics 53.8 (Aug. 2021). Publisher: Nature Publishing Group, pp. 1260–1269. ISSN: 1546-1718. DOI: 10.1038/s41588-021-00892-1. PMC8349845 34226706

[53] Andrés Aida M. “Targets of Balancing Selection in the Human Genome”. In: Molecular Biology and Evolution 26.12 (Dec. 2009), pp. 2755–2764. ISSN: 0737-4038. DOI: 10.1093/molbev/msp190. PMC2782326 19713326

[54] Booker Tom R., Jackson Benjamin C., and Keightley Peter D.. “Detecting positive selection in the genome”. In: BMC Biology 15.1 (Oct. 2017), p. 98. ISSN: 1741-7007. DOI: 10.1186/s12915-017-0434-y. PMC5662103 29084517

[55] Garbade Sven F. “Allelic phenotype values: a model for genotypebased phenotype prediction in phenylketonuria”. en. In: Genetics in Medicine 21.3 (Mar. 2019). Publisher: Nature Publishing Group, pp. 580–590. ISSN: 1530-0366. DOI: 10.1038/s41436-018-0081-x. 29997390

[56] Alfonso-Sánchez Miguel A. “An evolutionary approach to the high frequency of the Delta F508 CFTR mutation in European populations”. In: Medical Hypotheses 74.6 (June 2010), pp. 989–992. ISSN: 0306-9877. DOI: 10.1016/j.mehy.2009.12.018. 20110149

[57] Clark Andrew G.. “Mutation-selection balance with multiple alleles”. en. In: Mutation and Evolution. Springer, Dordrecht, 1998, pp. 41–47. ISBN: 978-94-011-5210-5. DOI: 10.1007/978-94-011-5210-5_4. 9720270

[58] Risch Neil “Geographic Distribution of Disease Mutations in the Ashkenazi Jewish Population Supports Genetic Drift over Selection”. In: The American Journal of Human Genetics 72.4 (Apr. 1, 2003), pp. 812–822. ISSN: 0002-9297, 1537-6605. DOI: 10.1086/373882. PMC1180346 12612865

[59] Oussalah Abderrahim “Population and evolutionary genetics of the PAH locus to uncover overdominance and adaptive mechanisms in phenylketonuria: Results from a multiethnic study”. English. In: eBioMedicine 51 (Jan. 2020). Publisher: Elsevier. ISSN: 2352-3964. DOI: 10.1016/j.ebiom.2019.102623. PMC7000351 31923802

[60] PCK2 maintains intestinal homeostasis and prevents colitis by protecting antibody-secreting cells from oxidative stress - Duan - 2024 - Immunology -Wiley Online Library. 10.1111/imm.13827 38934051

[61] Xue Shuaishuai “Dysregulation of phosphoenolpyruvate carboxykinase in cancers: A comprehensive analysis”. In: Cellular Signalling 120 (Aug. 2024), p. 111198. ISSN: 0898-6568. DOI: 10.1016/j.cellsig.2024.111198. 38697449

[62] Vincent Emma E. “Mitochondrial Phosphoenolpyruvate Carboxykinase Regulates Metabolic Adaptation and Enables Glucose-Independent Tumor Growth”. English. In: Molecular Cell 60.2 (Oct. 2015). Publisher: Elsevier, pp. 195–207. ISSN: 1097-2765. DOI: 10.1016/j.molcel.2015.08.013. 26474064

[63] Hemker Shelby L. “Saturation mapping of MUTYH variant effects using DNA repair reporters”. English. In: The American Journal of Human Genetics 112.9 (Sept. 2025). Publisher: Elsevier, pp. 2010–2026. ISSN: 0002-9297, 1537-6605. DOI: 10.1016/j.ajhg.2025.07.005. PMC12461019 40738107

[64] Young Candice L. A Maternal Germline Mutator Phenotype in a Family Affected by Heritable Colorectal Cancer. Dec. 18, 2023. DOI: 10.1101/2023.12.08.23299304. URL: https://www.medrxiv.org/content/10.1101/2023.12.08.23299304v1 (visited on 10/14/2024). Pre-published. PMC11631438 39403956

[65] Wilson P. D.. “Epithelial cell polarity and disease”. In: American Journal of Physiology-Renal Physiology 272.4 (Apr. 1997). Publisher: American Physiological Society, F434-F442. ISSN: 1931-857X. DOI: 10.1152/ajprenal.1997.272.4.F434. 9140043

[66] Lu Hui-Fei “Involvement and repair of epithelial barrier dysfunction in allergic diseases”. English. In: Frontiers in Immunology 15 (Feb. 2024). Publisher: Frontiers. ISSN: 1664-3224. DOI: 10.3389/fimmu.2024.1348272. PMC10867171 38361946

[67] Schleimer Robert P. and Berdnikovs Sergejs. “Etiology of epithelial barrier dysfunction in patients with type 2 inflammatory diseases”. English. In: Journal of Allergy and Clinical Immunology 139.6 (June 2017). Publisher: Elsevier, pp. 1752–1761. ISSN: 0091-6749, 1097-6825. DOI: 10.1016/j.jaci.2017.04.010. PMC5753806 28583447

[68] Hedrick Philip W.. “Balancing selection”. English. In: Current Biology 17.7 (Apr. 2007). Publisher: Elsevier, R230-R231. ISSN: 0960-9822. DOI: 10.1016/j.cub.2007.01.012. 17407748

[69] von Boehmer Harald. “Positive selection of lymphocytes”. In: Cell 76.2 (Jan. 1994), pp. 219–228. ISSN: 0092-8674. DOI: 10.1016/0092-8674(94)90330-1. 8293460

[70] Sironi Manuela “Evolutionary insights into host-pathogen interactions from mammalian sequence data”. en. In: Nature Reviews Genetics 16.4 (Apr. 2015). Publisher: Nature Publishing Group, pp. 224–236. ISSN: 1471-0064. DOI: 10.1038/nrg3905. PMC7096838 25783448

[71] Antonovics Janis “THE ORIGIN OF SPECIFICITY BY MEANS OF NATURAL SELECTION: EVOLVED AND NONHOST RESISTANCE IN HOST-PATHOGEN INTERACTIONS”. In: Evolution 67.1 (Jan. 2013), pp. 1–9. ISSN: 0014-3820. DOI: 10.1111/j.1558-5646.2012.01793.x. 23289557

[72] Lewontin R. C.. “POPULATION GENETICS”. In: Annual Review of Genetics 19 (Volume 19, 1985 Dec. 1, 1985), pp. 81–102. ISSN: 0066-4197, 1545-2948. DOI: 10.1146/annurev.ge.19.120185.000501. 3002243

[73] Crow J. F.. An Introduction to Population Genetics Theory. Scientific Publishers, Jan. 1, 2017. 609 pp. ISBN: 978-93-88148-06-1. Google Books: mpVEEAAAQBAJ.

[74] Ota Mineto Causal Modeling of Gene Effects from Regulators to Programs to Traits: Integration of Genetic Associations and Perturb-seq. Jan. 24, 2025. DOI: 10.1101/2025.01.22.634424. URL: https://www.biorxiv.org/content/10.1101/2025.01.22.634424v1(visited on 07/30/2025). Pre-published.

[75] Smith Courtney J “Integrative Analysis of Metabolite GWAS Illuminates the Molecular Basis of Pleiotropy and Genetic Correlation”. In: eLife 11 (Sept. 8, 2022). Ed. by Young Alexander et al., e79348. ISSN: 2050-084X. DOI: 10.7554/eLife.79348. PMC9536840 36073519

[76] Burton Paul R “Size Matters: Just How Big Is BIG?: Quantifying Realistic Sample Size Requirements for Human Genome Epidemiology”. In: International Journal of Epidemiology 38.1 (Feb. 1, 2009), pp. 263–273. ISSN: 0300-5771. DOI: 10.1093/ije/dyn147. PMC2639365 18676414

[77] White Jacob. “PubMed 2.0”. In: Medical Reference Services Quarterly 39.4 (Oct. 2020). Publisher: Routledge_eprint: https://doi.org/10.1080/02763869.2020.1826228, pp. 382–387. ISSN: 0276-3869. DOI: 10.1080/02763869.2020.1826228. 33085945

[78] AdamMargaret P. , eds. GeneReviews^®^. eng. Seattle (WA): University of Washington, Seattle, 1993.

[79] McLaren William “The Ensembl Variant Effect Predictor”. In: Genome Biology 17.1 (1 Dec. 2016), pp. 1–14. ISSN: 1474-760X. doi: 10.1186/s13059-016-0974-4. PMC4893825 27268795

[80] Molotkov Ivan, Mardis Elaine R., and Artomov Mykyta. “Making Sense of Missense: Challenges and Opportunities in Variant Pathogenicity Prediction”. In: Disease Models & Mechanisms 17.12 (Dec. 16, 2024), p. dmm052218. ISSN: 1754-8403. DOI: 10.1242/dmm.052218. PMC11683568 39676521

[81] Jackson Maria “The genetic basis of disease”. In: Essays in Biochemistry 62.5 (Dec. 2018), pp. 643–723. ISSN: 0071-1365. DOI: 10.1042/EBC20170053. PMC6279436 30509934

[82] Xiao Qingyang and Lauschke Volker M.. “The prevalence, genetic complexity and population-specific founder effects of human autosomal recessive disorders”. en. In: npj Genomic Medicine 6.1 (June 2021). Publisher: Nature Publishing Group, p. 41. ISSN: 2056-7944. DOI: 10.1038/s41525-021-00203-x. PMC8172936 34078906

[83] Landrum Melissa J “ClinVar: updates to support classifications of both germline and somatic variants”. In: Nucleic Acids Research 53.D1 (Jan. 2025), pp. D1313–D1321. ISSN: 1362-4962. DOI: 10.1093/nar/gkae1090. PMC11701624 39578691

[84] Castellani Carlo. “CFTR2: How will it help care?” In: Paediatric Respiratory Reviews. Royal Society of Medicine – The 26th symposium: Cystic fibrosis in children and adults, 20th November 201214 (May 2013), pp. 2–5. ISSN: 1526-0542. DOI: 10.1016/j.prrv.2013.01.006.

[85] Kumar Mukesh “WilsonGen a comprehensive clinically annotated genomic variant resource for Wilson's Disease”. en. In: Scientific Reports 10.1 (June 2020). Publisher: Nature Publishing Group, p. 9037. ISSN: 2045-2322. DOI: 10.1038/s41598-020-66099-2. PMC7270127 32493955

[86] Nagral Aabha. “Gaucher Disease”. In: Journal of Clinical and Experimental Hepatology 4.1 (Mar. 2014), pp. 37–50. ISSN: 0973-6883. DOI: 10.1016/j.jceh.2014.02.005. PMC4017182 25755533

[87] CastiglioneAlessandro and MöllerClaes. “Usher Syndrome”. en. In: Audiology Research 12.1 (Feb. 2022). Publisher: Multidisciplinary Digital Publishing Institute, pp. 42–65. ISSN: 2039-4349. DOI: 10.3390/audiolres12010005.

[88] Mercuri Eugenio “Spinal muscular atrophy”. en. In: Nature Reviews Disease Primers 8.1 (Aug. 2022). Publisher: Nature Publishing Group, p. 52. ISSN: 2056-676X. DOI: 10.1038/s41572-022-00380-8. 35927425

[89] Haller Benjamin C. and Messer Philipp W.. “SLiM 4: Multispecies Eco-Evolutionary Modeling”. In: The American Naturalist 201.5 (May 2023). Publisher: The University of Chicago Press, E127-E139. ISSN: 0003-0147. DOI: 10.1086/723601. PMC10793872 37130229

[90] Mbatchou Joelle “Computationally efficient whole-genome regression for quantitative and binary traits”. en. In: Nature Genetics 53.7 (July 2021). Publisher: Nature Publishing Group, pp. 1097–1103. ISSN: 1546-1718. DOI: 10.1038/s41588-021-00870-7. 34017140

[91] Bycroft Clare “The UK Biobank resource with deep phenotyping and genomic data”. en. In: Nature 562.7726 (Oct. 2018). Publisher: Nature Publishing Group, pp. 203–209. ISSN: 1476-4687. DOI: 10.1038/s41586-018-0579-z. PMC6786975 30305743

[92] Liu Yaowu “ACAT: A Fast and Powerful p Value Combination Method for Rare-Variant Analysis in Sequencing Studies”. In: The American Journal of Human Genetics 104.3 (Mar. 7, 2019), pp. 410–421. ISSN:0002-9297. DOI: 10.1016/j.ajhg.2019.01.002. PMC6407498 30849328

[93] Ashburner Michael “Gene Ontology: Tool for the Unification of Biology”. In: Nature Genetics 25.1 (May 2000), pp. 25–29. ISSN: 1546-1718. DOI: 10.1038/75556. PMC3037419 10802651

[94] The Gene Ontology Consortium et al. “The Gene Ontology Knowledgebase in 2023”. In: Genetics 224.1 (May 2, 2023), iyad031. ISSN: 1943-2631. DOI: 10.1093/genetics/iyad031. PMC10158837 36866529

[95] Karlin Samuel and Taylor Howard E. A second course in stochastic processes. Elsevier, 1981.

[96] Nei Masatoshi. “The frequency distribution of lethal chromosomes in finite populations.” In: Proceedings of the National Academy of Sciences 60.2 (1968), pp. 517–524. 10.1073/pnas.60.2.517 PMC225078 5248809

[97] Schraiber Joshua G, Spence Jeffrey P, and Edge Michael D. “Estimation of demography and mutation rates from one million haploid genomes”. In: bioRxiv (2024), pp. 2024–09. 10.1016/j.ajhg.2025.07.008 PMC12461025 40812314

[98] The mpmath development team. mpmath: a Python library for arbitraryprecision floating-point arithmetic (version 1.3.0). http://mpmath.org/. 2023.

[99] Spence Jeffrey P “Scaling the discrete-time Wright–Fisher model to biobank-scale datasets”. In: Genetics 225.3 (2023), iyad168. 10.1093/genetics/iyad168 PMC10627256 37724741

[100] Bender Carl M and Orszag Steven A. Advanced mathematical methods for scientists and engineers I: Asymptotic methods and perturbation theory. Springer Science & Business Media, 2013.

