# Supplementary material for: Allele Frequencies at Recessive Disease Genes are Mainly Determined by Pleiotropic Effects in Heterozygotes
Source: bioRxiv. 2025 Dec 9:2025.12.05.692665. Preprint. [Version 1] doi: 10.64898/2025.12.05.692665 (PMC12822701; doi:10.64898/2025.12.05.692665)
Supplement: Supplementary file 1 [file NIHPP2025.12.05.692665V1-supplement-1.pdf]

bioRxiv preprint doi: https://doi.org/10.64898/2025.12.05.692665; this version posted December 9, 2025. The copyright holder for this preprint (which was not certified by peer review) is the author/funder, who has granted bioRxiv a license to display the preprint in perpetuity. It is made available under a CC-BY 4.0 International license.

Supplemental Information

Contents

List of Supplemental Figures **30**

List of Supplemental Tables **38**

Appendix **47**

bioRxiv preprint doi: https://doi.org/10.64898/2025.12.05.692665; this version posted December 9, 2025. The copyright holder for this preprint (which was not certified by peer review) is the author/funder, who has granted bioRxiv a license to display the preprint in perpetuity. It is made available under a CC-BY 4.0 International license.

List of Supplemental Figures

S1 **LoF and Pathogenic Variation:**
A) Density plot for observed relationship between square root mutational target size and p_{LoF} for recessive genes using NFE allele frequencies. **B)** Proportional split of observed pathogenic variation for 25 commonly-studied recessive disease genes based on predicted molecular consequence (LoF, Frameshift, Missense, or Other). **C)** Percentage of observed pathogenic variation for 25 commonly-studied recessive disease genes predicted to be LoFs. Bands represent two standard error confidence intervals surrounding LoF percentage point estimate. Red line represents average percentage of pathogenic variation predicted to be LoFs between all recessive disease genes. 32

S2 **Other Population Mutation-Selection Balance:**
A) Observed relationship between square root mutational target and p_{path} for recessive genes using NFE allele frequencies. **B)** Observed relationship between square root mutational target and p_{path} for recessive genes using FIN allele frequencies. **C)** Observed relationship between square root mutational target and p_{path} for recessive genes using AFR allele frequencies. **D)** Observed relationship between square root mutational target and p_{path} for recessive genes using SAS allele frequencies. 33

S3 **Mutation Selection Balance for Recessive Lethal Genes:**
A) Observed relationship between square root mutational target size and p_{path} for recessive lethal disease genes. Genes with a predicted p_{path} in the expected range for recessive mutation-selection balance are labeled using non-Finnish European (NFE) allele frequencies. **B)** Observed relationship between square root mutation target size and p_{path} for recessive genes using NFE allele frequencies and not excluding LoF variants with a frequency greater than 0.005 prior to calculating p_{path}. 34

S4 **Inbreeding Models:**
A) Percentage of recessive disease genes that fit the classic recessive mutation-selection balance for different levels of population inbreeding. **B)** Comparison of the classic recessive mutation-selection balance model and adjusted Nei model (Equation 9) for a finite population for populations with no inbreeding. Lines represent predicted p_{path} for a given mutational target size for each model assuming recessive lethality. Dots represent simulated p_{path} data for recessive lethal genes with varying mutational target sizes. **C)** Observed relationship between square root mutational target size and p_{path} for recessive genes using NFE allele frequencies. Lines represent the expected relationship between square root mutational target size and p_{path} under finite population model for different levels of inbreeding in the population. **D)** Percentage of recessive disease genes that fit the finite population model for different levels of population inbreeding. 35

bioRxiv preprint doi: https://doi.org/10.64898/2025.12.05.692665; this version posted December 9, 2025. The copyright holder for this preprint (which was not certified by peer review) is the author/funder, who has granted bioRxiv a license to display the preprint in perpetuity. It is made available under a CC-BY 4.0 International license.

S5 **Heterozygous Effects:**
A) Empirical cumulative distribution function of ACAT omnibus test of p-values from LoF burden tests for 161 UK BioBank (UKB) quantitative traits across 796 recessive disease genes. The vertical line represents p-values that surpass 0.05. **B)** Empirical cumulative distribution function of ACAT omnibus test of p-values from LoF burden tests for 21 uncorrelated UKB quantitative traits across 796 recessive disease genes. The vertical line represents p-values that surpass 0.05. **C)** Empirical cumulative distribution function of ACAT omnibus test of p-values from LoF burden tests for 161 UK BioBank (UKB) quantitative traits across a random sample of 796 non-recessive disease genes. The vertical line represents p-values that surpass 0.05. **D)** Empirical cumulative distribution function of ACAT omnibus test of p-values from LoF burden tests for 21 uncorrelated UKB quantitative traits across a random sample of 796 non-recessive disease genes. The vertical line represents p-values that surpass 0.05. **E)** Quantile-quantile plot of 21 LoF burden test p-values from uncorrelated traits for the recessive *PCSK1* gene. **F)** Quantile-quantile plot of 21 LoF burden test p-values from uncorrelated traits for the recessive *EPX* gene. 36
S6 **Mutation-Selection Balance Under Balancing Selection:**
A) Observed relationship between square root mutational target and p_{path} for recessive disease genes previously thought to be affected by balancing selection using NFE allele frequencies. **B)** Observed relationship between square root mutational target and p_{path} for recessive disease genes previously thought to be affected by balancing selection using FIN allele frequencies. **C)** Observed relationship between square root mutational target and p_{path} for recessive disease genes previously thought to be affected by balancing selection using AFR allele frequencies. **D)** Observed relationship between square root mutational target and p_{path} for recessive disease genes previously thought to be affected by balancing selection using SAS allele frequencies. 37

bioRxiv preprint doi: https://doi.org/10.64898/2025.12.05.692665; this version posted December 9, 2025. The copyright holder for this preprint (which was not certified by peer review) is the author/funder, who has granted bioRxiv a license to display the preprint in perpetuity. It is made available under a CC-BY 4.0 International license.

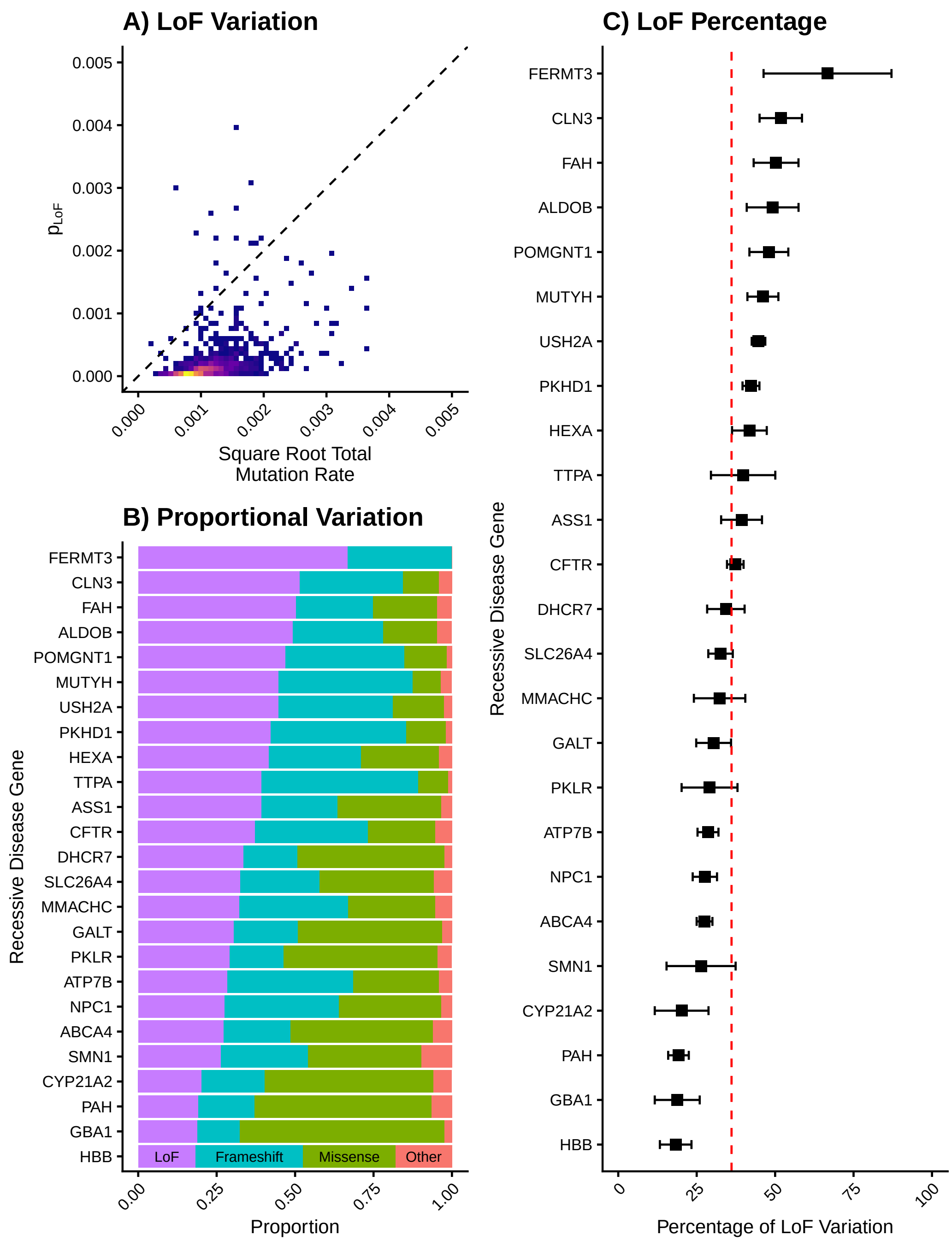

Figure S1: LoF and Pathogenic Variation:
A) Density plot for observed relationship between square root mutational target size and p_{LoF} for recessive genes using NFE allele frequencies. **B)** Proportional split of observed pathogenic variation for 25 commonly-studied recessive disease genes based on predicted molecular consequence (LoF, Frameshift, Missense, or Other). **C)** Percentage of observed pathogenic variation for 25 commonly-studied recessive disease genes predicted to be LoFs. Bands represent two standard error confidence intervals surrounding LoF percentage point estimate. Red line represents average percentage of pathogenic variation predicted to be LoFs between all recessive disease genes.

bioRxiv preprint doi: https://doi.org/10.64898/2025.12.05.692665; this version posted December 9, 2025. The copyright holder for this preprint (which was not certified by peer review) is the author/funder, who has granted bioRxiv a license to display the preprint in perpetuity. It is made available under a CC-BY 4.0 International license.

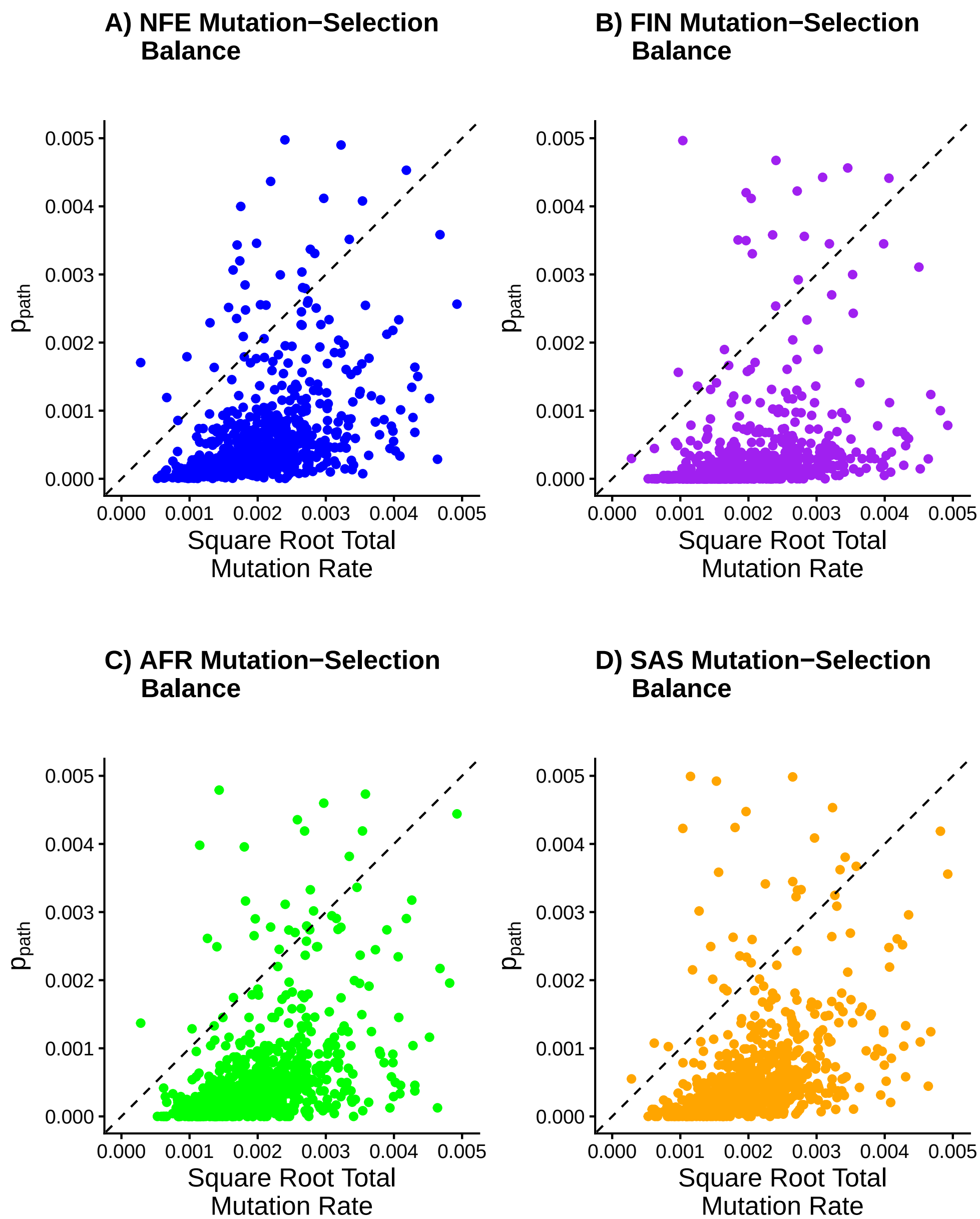

Figure S2: Other Population Mutation-Selection Balance:
A) Observed relationship between square root mutational target and p_{path} for recessive genes using NFE allele frequencies. **B)** Observed relationship between square root mutational target and p_{path} for recessive genes using FIN allele frequencies. **C)** Observed relationship between square root mutational target and p_{path} for recessive genes using AFR allele frequencies. **D)** Observed relationship between square root mutational target and p_{path} for recessive genes using SAS allele frequencies.

bioRxiv preprint doi: https://doi.org/10.64898/2025.12.05.692665; this version posted December 9, 2025. The copyright holder for this preprint (which was not certified by peer review) is the author/funder, who has granted bioRxiv a license to display the preprint in perpetuity. It is made available under a CC-BY 4.0 International license.

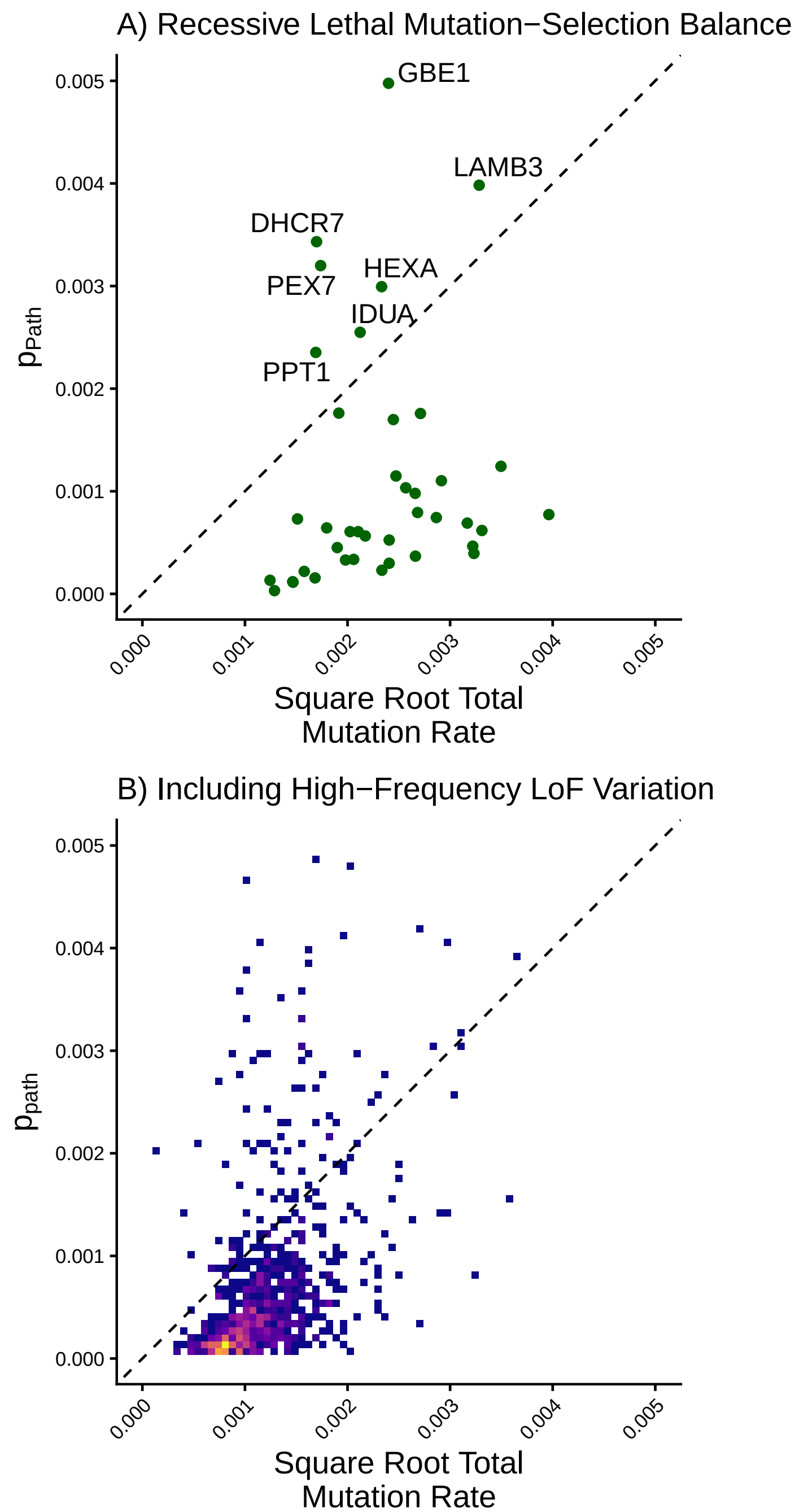

Figure S3: Mutation Selection Balance for Recessive Lethal Genes:
A) Observed relationship between square root mutational target size and p_{path} for recessive lethal disease genes. Genes with a predicted p_{path} in the expected range for recessive mutation-selection balance are labeled using non-Finnish European (NFE) allele frequencies. **B)** Observed relationship between square root mutation target size and p_{path} for recessive genes using NFE allele frequencies and not excluding LoF variants with a frequency greater than 0.005 prior to calculating p_{path}.

bioRxiv preprint doi: https://doi.org/10.64898/2025.12.05.692665; this version posted December 9, 2025. The copyright holder for this preprint (which was not certified by peer review) is the author/funder, who has granted bioRxiv a license to display the preprint in perpetuity. It is made available under a CC-BY 4.0 International license.

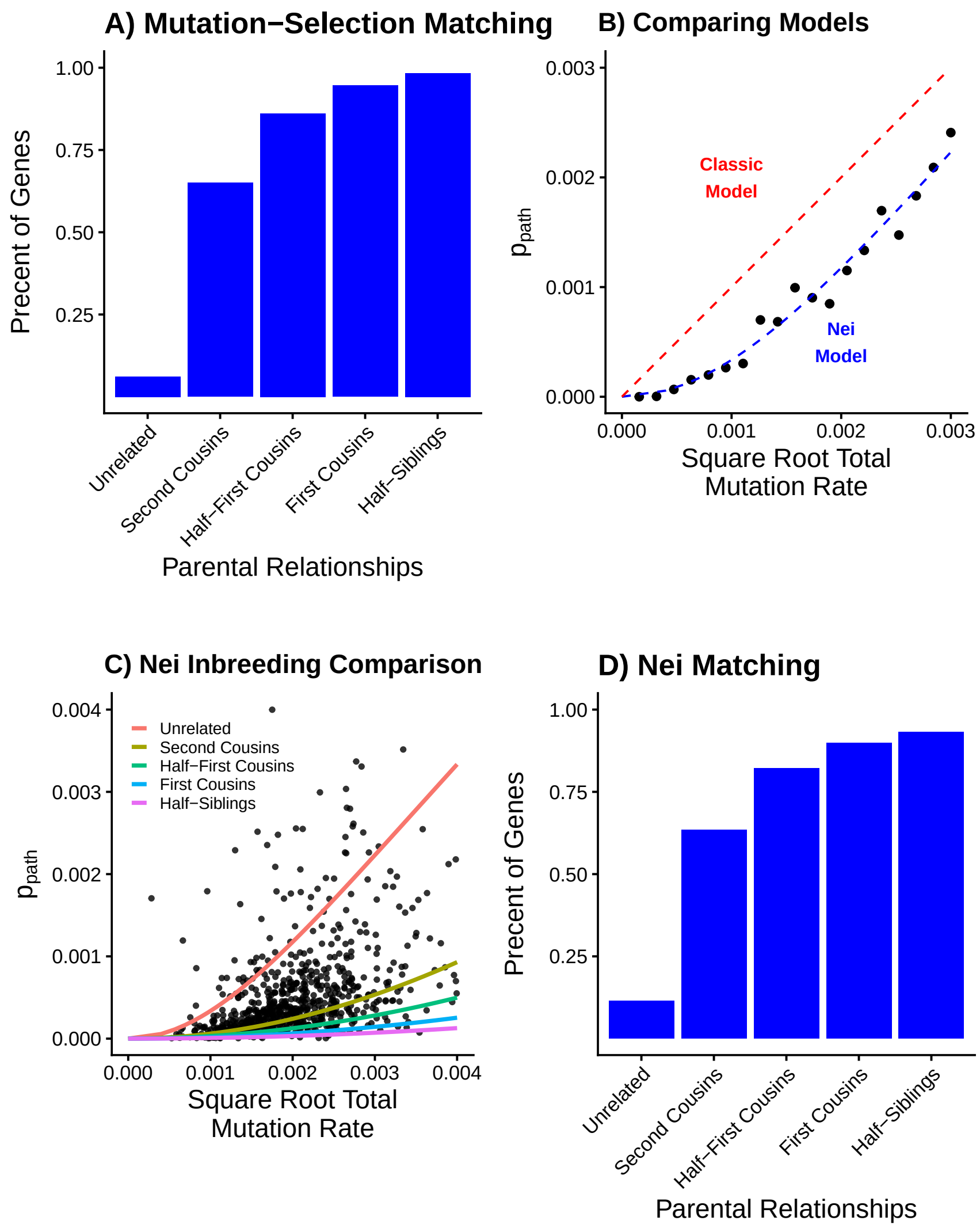

Figure S4: Inbreeding Models:
A) Percentage of recessive disease genes that fit the classic recessive mutation-selection balance for different levels of population inbreeding. **B)** Comparison of the classic recessive mutation-selection balance model and adjusted Nei model (Equation 9) for a finite population for populations with no inbreeding. Lines represent predicted p_{path} for a given mutational target size for each model assuming recessive lethality. Dots represent simulated p_{path} data for recessive lethal genes with varying mutational target sizes. **C)** Observed relationship between square root mutational target size and p_{path} for recessive genes using NFE allele frequencies. Lines represent the expected relationship between square root mutational target size and p_{path} under finite population model for different levels of inbreeding in the population. **D)** Percentage of recessive disease genes that fit the finite population model for different levels of population inbreeding.

bioRxiv preprint doi: https://doi.org/10.64898/2025.12.05.692665; this version posted December 9, 2025. The copyright holder for this preprint (which was not certified by peer review) is the author/funder, who has granted bioRxiv a license to display the preprint in perpetuity. It is made available under a CC-BY 4.0 International license.

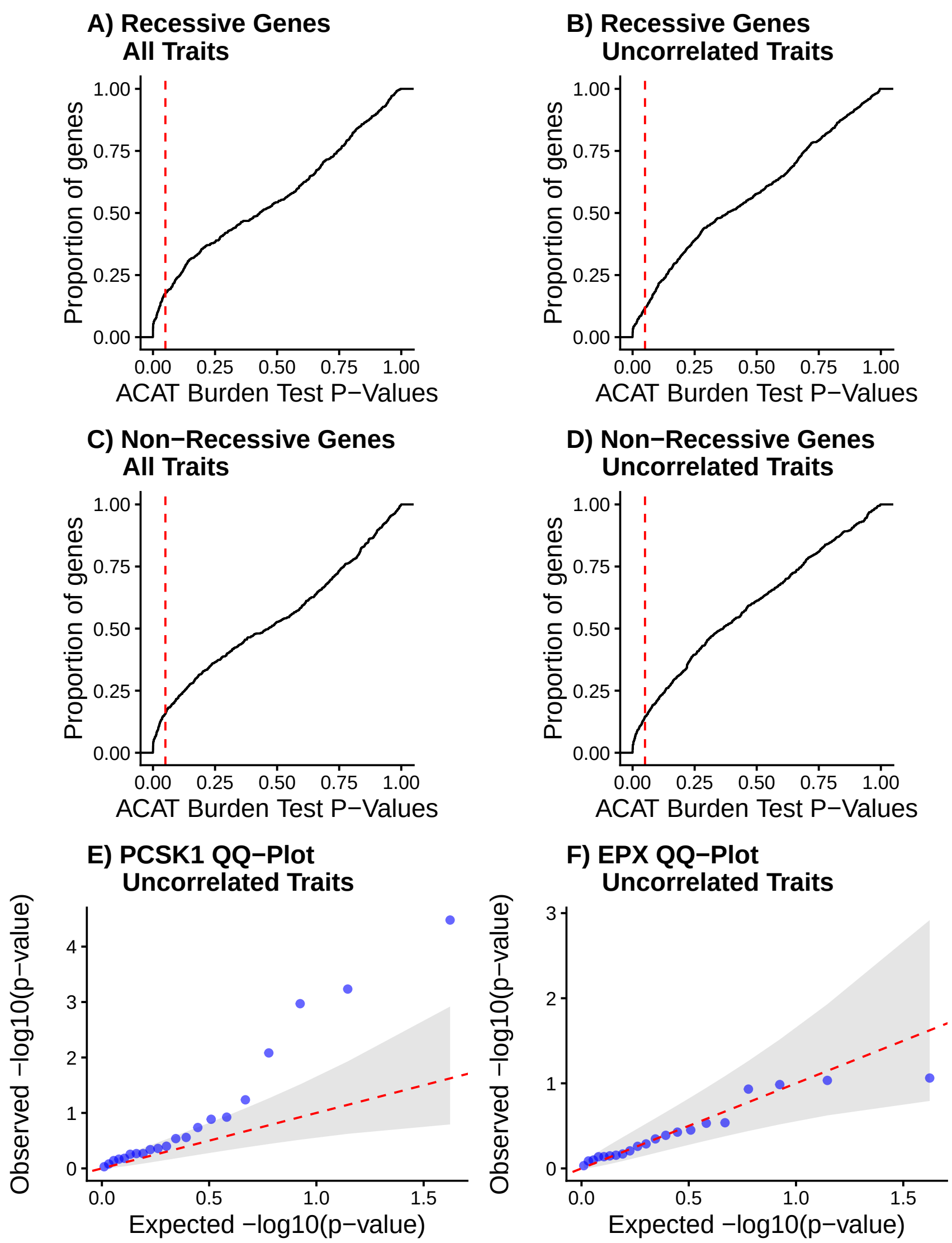

Figure S5: Heterozygous Effects:
A) Empirical cumulative distribution function of ACAT omnibus test of p-values from LoF burden tests for 161 UK BioBank (UKB) quantitative traits across 796 recessive disease genes. The vertical line represents p-values that surpass 0.05. **B)** Empirical cumulative distribution function of ACAT omnibus test of p-values from LoF burden tests for 21 uncorrelated UKB quantitative traits across 796 recessive disease genes. The vertical line represents p-values that surpass 0.05. **C)** Empirical cumulative distribution function of ACAT omnibus test of p-values from LoF burden tests for 161 UK BioBank (UKB) quantitative traits across a random sample of 796 non-recessive disease genes. The vertical line represents p-values that surpass 0.05. **D)** Empirical cumulative distribution function of ACAT omnibus test of p-values from LoF burden tests for 21 uncorrelated UKB quantitative traits across a random sample of 796 non-recessive disease genes. The vertical line represents p-values that surpass 0.05. **E)** Quantile-quantile plot of 21 LoF burden test p-values from uncorrelated traits for the recessive *PCSK1* gene. **F)** Quantile-quantile plot of 21 LoF burden test p-values from uncorrelated traits for the recessive *EPX* gene.

bioRxiv preprint doi: https://doi.org/10.64898/2025.12.05.692665; this version posted December 9, 2025. The copyright holder for this preprint (which was not certified by peer review) is the author/funder, who has granted bioRxiv a license to display the preprint in perpetuity. It is made available under a CC-BY 4.0 International license.

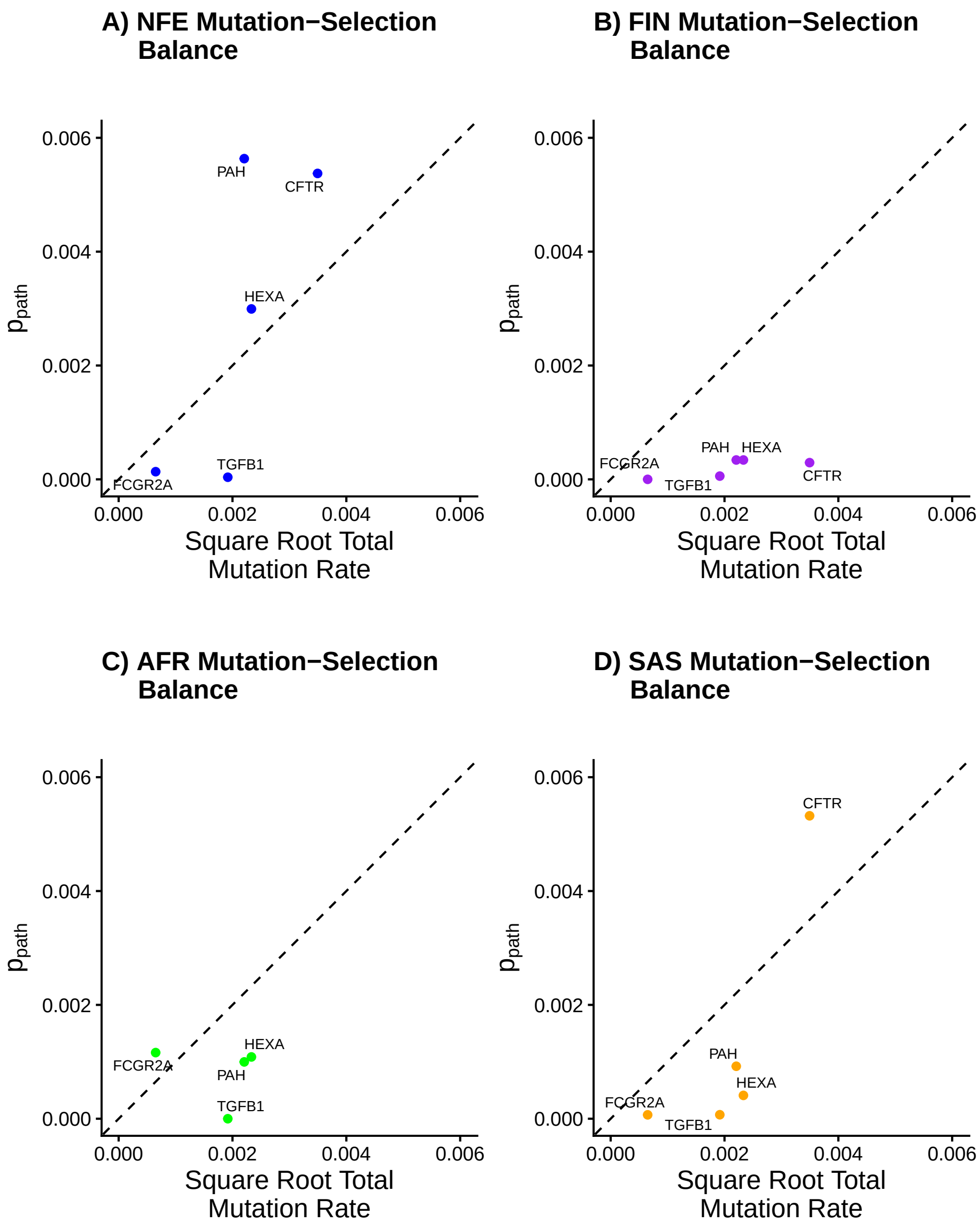

Figure S6: Mutation-Selection Balance Under Balancing Selection:
A) Observed relationship between square root mutational target and p_{path} for recessive disease genes previously thought to be affected by balancing selection using NFE allele frequencies. **B)** Observed relationship between square root mutational target and p_{path} for recessive disease genes previously thought to be affected by balancing selection using FIN allele frequencies. **C)** Observed relationship between square root mutational target and p_{path} for recessive disease genes previously thought to be affected by balancing selection using AFR allele frequencies. **D)** Observed relationship between square root mutational target and p_{path} for recessive disease genes previously thought to be affected by balancing selection using SAS allele frequencies.

bioRxiv preprint doi: https://doi.org/10.64898/2025.12.05.692665; this version posted December 9, 2025. The copyright holder for this preprint (which was not certified by peer review) is the author/funder, who has granted bioRxiv a license to display the preprint in perpetuity. It is made available under a CC-BY 4.0 International license.

List of Supplemental Tables

S1 **Recessive Disease Genes:**
List of 796 genes associated with only recessive diseases and no other documented inheritance method listed (in OMIM as of December 2024) (Full Table in Separate File) 39

S2 **Classification of Pathogenic/Likely Pathogenic Variation for Studied Recessive Disease Genes:**
List of 25 commonly studied recessive diseases and genes associated with those diseases. For each gene, the number of pathogenic/likely pathogenic variants (identified through ClinVar) is listed as well as the percentage of this variation that is predicted to be loss-of-function (LoF) (nonsense, splice-acceptor disrupting, or splice-donor discrupting), frameshift, missense, or other. For each gene, a conversion factor to account for missing non-LoF pathogenic variation is included and calculated as the inverse and square root inverse of the percentage of LoF variation, respectively. Other variation defined as UTR variants, genic upstream/downstream transcript variants, inframe indels, intron variants, non-coding transcript variants, and synonymous variants . . 40

S3 **PCSK1 LoF Burden Tests:**
PCKS1 LoF burden test results for 161 non-disease quantitative traits from the UK Biobank. Each row includes effect size estimate, standard error, and p-value for association between PCSK1 LoFs and phenotype (Full Table in Separate File) 41

S4 **Potentially Purely Recessive Selection Genes Across Populations:**
List of recessive genes that appear to follow classic recessive mutation-selection balance based on at least one population-specific pLoF being larger than the gene's square root mutational target size. Square root mutational target size, pLoFs for Non-Finnish European, Finnish, African, and South Asian populations, and populations where the gene matches mutation-selection balance model are included for each gene. 41

S5 **Select Recessive Genes GO Enrichment:**
Gene Ontology enrichment of recessive disease genes in the expected range for recessive mutation-selection balance based on pLoF in Non-Finnish European, Finnish, African, and South Asian populations. Each row identifies the enrichment of purely recessive genes in biological, molecular, and cellular functions and the significance of this enrichment. Background gene list is all recessive disease genes. 45

bioRxiv preprint doi: https://doi.org/10.64898/2025.12.05.692665; this version posted December 9, 2025. The copyright holder for this preprint (which was not certified by peer review) is the author/funder, who has granted bioRxiv a license to display the preprint in perpetuity. It is made available under a CC-BY 4.0 International license.

Table S1: Recessive Disease Genes:
List of 796 genes associated with only recessive diseases and no other documented inheritance method listed (in OMIM as of December 2024)
(Full Table in Separate File)

Table S2: Classification of Pathogenic/Likely Pathogenic Variation for Studied Recessive Disease Genes: List of 25 commonly studied recessive diseases and genes associated with those diseases. For each gene, the number of pathogenic/likely pathogenic variants (identified through ClinVar) is listed as well as the percentage of this variation that is predicted to be loss-of-function (LoF) (nonsense, splice-acceptor disrupting, or splice-donor discrupting), frameshift, missense, or other. For each gene, a conversion factor to account for missing non-LoF pathogenic variation is included and calculated as the inverse and square root inverse of the percentage of LoF variation, respectively. Other variation defined as UTR variants, genic upstream/downstream transcript variants, inframe indels, intron variants, non-coding transcript variants, and synonymous variants

Genes	Associated_Disease	n_variants	pct_LoF	pct_Frameshift	pct_Missense	pct_Other	pLoF_conversion	sqrtMT_conversion
ABCA4	Stargardt disease	1380	27.5	21.4	45.70	6.14	3.63	1.91
ALDOB	Hereditary fructose intolerance	128	49.2	28.9	17.20	4.69	2.03	1.43
ASS1	Citrullinemia type I	206	39.3	24.3	33.00	3.40	2.54	1.59
ATP7B	Wilson disease	784	28.6	40.2	27.40	4.21	3.50	1.87
CFTR	Cystic fibrosis	1250	37.3	36.1	21.50	5.36	2.68	1.64
CLN3	Juvenile neuronal ceroid lipofuscinosis	191	51.8	33.0	11.50	4.19	1.93	1.39
CYP21A2	Congenital adrenal hyperplasia	119	20.2	20.2	53.80	5.88	4.96	2.23
DHCR7	Smith-Lemli-Opitz syndrome	245	34.3	17.6	47.80	2.45	2.92	1.71
FAH	Tyrosinemia type I	171	50.3	24.6	20.50	4.68	1.99	1.41
FERMT3	Leukocyte adhesion deficiency type III	21	66.7	33.3	0.00	0.00	1.50	1.22
GALT	Classic galactosemia	283	30.4	20.5	45.90	3.18	3.29	1.81
GBA1	Gaucher disease	170	18.8	13.5	65.30	2.35	5.31	2.30
HBB	Sickle cell disease	344	18.3	34.3	29.40	18.00	5.46	2.34
HEXA	Tay-sachs disease	285	41.8	29.5	24.90	4.21	2.39	1.55
MMACHC	Cobalamin C deficiency	130	32.3	34.6	27.70	5.38	3.10	1.76
MUTYH	MUTYH-associated polyposis	358	46.1	44.1	9.22	3.63	2.17	1.47
NPC1	Niemann-Pick disease, type C1	580	27.6	36.4	32.60	3.45	3.63	1.90
PAH	Phenylketonuria (PKU)	808	19.2	17.8	56.40	6.56	5.21	2.28
PKHD1	Autosomal recessive polycystic kidney disease	1200	42.3	43.1	12.70	1.92	2.36	1.54
PKLR	Pyruvate kinase deficiency	110	29.1	17.3	49.10	4.55	3.44	1.85
POMGNT1	Congenital muscular dystrophy	227	48.0	38.8	13.70	1.76	2.08	1.44
SLC26A4	Pendred syndrome, DFNB4 deafness	574	32.6	25.3	36.40	5.75	3.07	1.75
SMN1	Spinal muscular atrophy	72	26.4	27.8	36.10	9.72	3.79	1.95
TTPA	Ataxia with vitamin E deficiency	83	39.8	50.6	9.64	1.20	2.52	1.59
USH2A	Usher syndrome type II	1860	44.7	36.4	16.30	2.59	2.23	1.49

bioRxiv preprint doi: https://doi.org/10.64898/2025.12.05.692665; this version posted December 9, 2025. The copyright holder for this preprint (which was not certified by peer review) is the author/funder, who has granted bioRxiv a license to display the preprint in perpetuity. It is made available under a CC-BY 4.0 International license.

bioRxiv preprint doi: https://doi.org/10.64898/2025.12.05.692665; this version posted December 9, 2025. The copyright holder for this preprint (which was not certified by peer review) is the author/funder, who has granted bioRxiv a license to display the preprint in perpetuity. It is made available under a CC-BY 4.0 International license.

Table S3: PCSK1 LoF Burden Tests:
PCKS1 LoF burden test results for 161 non-disease quantitative traits from the UK Biobank. Each row includes effect size estimate, standard error, and p-value for association between PCSK1 LoFs and phenotype
(Full Table in Separate File)

Table S4: Potentially Purely Recessive Selection Genes Across Populations: List of recessive genes that appear to follow classic recessive mutation-selection balance based on at least one population-specific pLoF being larger than the gene's square root mutational target size. Square root mutational target size, pLoFs for Non-Finnish European, Finnish, African, and South Asian populations, and populations where the gene matches mutation-selection balance model are included for each gene.

Gene	Square Root Mutational Target Size	Non-Finnish pPath	Finnish pPath	African pPath	South Asian pPath	Potentially Recessive Populations
ABCC2	2.04e-03	4.08e-03	2.43e-03	4.19e-03	7.17e-03	NFE, FIN, AFR, SAS
ABCG5	1.14e-03	1.18e-03	1.17e-03	5.40e-04	2.34e-03	NFE, FIN, SAS
ABCG8	1.26e-03	4.37e-03	8.60e-03	2.78e-03	1.37e-03	NFE, FIN, AFR, SAS
ACADSB	1.05e-03	2.48e-03	1.98e-04	3.16e-03	7.23e-04	NFE, AFR
ADAMTSL4	1.82e-03	6.41e-04	2.45e-04	2.91e-03	7.55e-04	AFR
AGL	2.24e-03	2.12e-03	7.77e-04	2.74e-03	9.92e-04	AFR
AGPAT2	8.26e-04	1.06e-04	0.00e+00	4.79e-03	1.02e-04	AFR
AHI1	1.86e-03	1.85e-03	2.70e-03	2.78e-03	1.69e-03	FIN, AFR
ALG12	1.03e-03	5.61e-04	1.22e-03	3.73e-04	3.41e-04	FIN
ALMS1	1.55e-03	2.79e-03	9.76e-04	2.37e-03	3.23e-03	NFE, AFR, SAS
ALOXE3	1.47e-03	1.22e-03	1.26e-03	6.65e-04	1.54e-03	SAS
ANO6	1.80e-03	1.85e-03	4.86e-04	1.16e-03	1.47e-03	NFE
AP5Z1	1.52e-03	2.26e-03	1.54e-04	1.58e-03	1.35e-03	NFE, AFR
APTX	1.09e-03	1.70e-03	4.86e-05	9.17e-04	3.07e-04	NFE
ARSA	1.14e-03	3.46e-03	1.58e-03	7.06e-04	7.28e-04	NFE, FIN
ASPM	2.97e-03	3.43e-03	2.68e-03	3.04e-03	2.67e-03	NFE, AFR
ATP7B	1.53e-03	1.56e-03	2.04e-03	8.71e-04	3.45e-03	NFE, FIN, SAS
ATPAF2	7.89e-04	1.24e-04	0.00e+00	1.12e-03	6.86e-05	AFR
AUH	1.06e-03	3.81e-04	3.51e-03	2.08e-04	4.14e-04	FIN
BBS2	1.65e-03	2.51e-03	2.33e-03	7.05e-04	6.83e-04	NFE, FIN
BLM	1.52e-03	2.45e-03	6.33e-04	1.33e-03	1.41e-03	NFE
CARD9	1.13e-03	1.98e-04	3.50e-03	2.90e-03	4.48e-03	FIN, AFR, SAS
CATSPER1	9.47e-04	3.32e-04	4.95e-05	1.74e-03	4.11e-04	AFR
CC2D2A	2.45e-03	1.34e-03	6.89e-04	3.18e-03	2.52e-03	AFR, SAS
CD36	1.01e-03	4.00e-03	9.72e-05	7.60e-03	1.11e-02	NFE, AFR, SAS
CD3D	4.74e-04	4.01e-04	0.00e+00	2.49e-04	1.02e-03	SAS
CD3G	6.65e-04	2.84e-04	7.87e-04	2.08e-04	1.02e-04	FIN
CD8A	6.90e-04	1.05e-04	0.00e+00	4.16e-05	7.87e-04	SAS
CEP152	2.06e-03	2.55e-03	3.92e-04	4.73e-03	3.67e-03	NFE, AFR, SAS
CEP290	2.77e-03	5.09e-03	1.00e-03	1.96e-03	4.19e-03	NFE, SAS
CERKL	1.17e-03	2.56e-03	4.12e-03	9.56e-04	2.26e-03	NFE, FIN, SAS
CHRNG	1.35e-03	5.97e-04	1.31e-03	4.56e-04	1.71e-03	SAS
CNGA1	1.37e-03	1.54e-03	1.46e-04	6.65e-04	8.61e-04	NFE
CNGB1	1.68e-03	1.94e-03	2.92e-04	5.89e-03	1.61e-03	NFE, AFR
CNGB3	1.47e-03	8.80e-04	3.89e-04	2.70e-03	6.51e-04	AFR
COG5	1.61e-03	6.35e-04	1.46e-04	5.41e-04	7.35e-03	SAS
COX15	1.14e-03	1.76e-03	1.46e-04	8.72e-04	5.81e-04	NFE
COX4I2	7.22e-04	2.85e-04	1.36e-03	3.32e-04	1.72e-04	FIN
CSTB	3.83e-04	1.19e-03	0.00e+00	2.07e-04	0.00e+00	NFE
CUBN	2.59e-03	5.70e-03	3.11e-03	1.22e-02	6.83e-03	NFE, FIN, AFR, SAS
CUL7	2.15e-03	8.33e-04	1.54e-04	2.45e-03	9.63e-04	AFR
CYP27A1	1.29e-03	1.31e-03	1.46e-04	1.45e-03	3.41e-03	NFE, AFR, SAS

Continued on next page

bioRxiv preprint doi: https://doi.org/10.64898/2025.12.05.692665; this version posted December 9, 2025. The copyright holder for this preprint (which was not certified by peer review) is the author/funder, who has granted bioRxiv a license to display the preprint in perpetuity. It is made available under a CC-BY 4.0 International license.

Gene	Square Root Mutational Target Size	Non-Finnish pPath	Finnish pPath	African pPath	South Asian pPath	Potentially Recessive Populations
CYP2C19	8.07e-04	7.43e-04	6.32e-04	2.49e-03	1.12e-02	AFR, SAS
CYP2R1	1.17e-03	4.85e-04	1.47e-04	1.30e-03	8.04e-04	AFR
CYP4F22	1.18e-03	2.03e-04	3.30e-03	4.57e-04	1.71e-04	FIN
CYP4V2	1.13e-03	6.80e-04	4.20e-03	4.98e-04	3.42e-04	FIN
D2HGDH	1.01e-03	1.43e-04	4.92e-04	1.04e-03	3.76e-04	AFR
DARS2	1.41e-03	9.90e-04	5.39e-03	1.37e-03	5.46e-04	FIN
DBT	1.12e-03	1.19e-04	4.86e-05	2.65e-03	1.37e-04	AFR
DCLRE1C	1.15e-03	6.54e-04	1.46e-04	1.87e-03	3.76e-04	AFR
DDB2	1.17e-03	2.79e-04	1.60e-03	4.15e-05	3.41e-05	FIN
DHCR7	9.78e-04	3.43e-03	6.52e-03	5.44e-03	1.20e-03	NFE, FIN, AFR, SAS
DMGDH	1.56e-03	6.85e-03	4.22e-03	2.57e-03	8.81e-03	NFE, FIN, AFR, SAS
DNAAF1	1.63e-04	1.71e-03	2.97e-04	1.37e-03	5.51e-04	NFE, FIN, AFR, SAS
DNAH11	3.61e-03	4.93e-03	3.07e-03	3.42e-03	5.71e-03	NFE, SAS
DNAH5	3.65e-03	3.34e-03	7.82e-03	3.20e-03	3.18e-03	FIN
DNAI1	1.33e-03	1.82e-03	6.80e-04	9.54e-04	2.73e-04	NFE
DNAI2	1.39e-03	7.31e-04	1.96e-04	1.78e-03	2.22e-03	AFR, SAS
DPYD	1.65e-03	1.63e-02	2.44e-04	2.49e-03	1.33e-02	NFE, AFR, SAS
DPYS	1.21e-03	1.78e-03	9.71e-05	4.98e-04	8.88e-04	NFE
DSG4	1.55e-03	7.07e-04	1.95e-04	4.19e-03	6.85e-04	AFR
DUOX2	1.93e-03	3.52e-03	3.89e-04	3.82e-03	3.62e-03	NFE, AFR, SAS
DUOXA2	7.54e-04	5.46e-04	9.83e-05	1.04e-03	7.53e-04	AFR
DYSF	2.50e-03	1.50e-03	5.90e-04	6.15e-03	2.96e-03	AFR, SAS
EIF2B1	8.99e-04	8.33e-04	4.86e-05	7.05e-04	3.58e-03	SAS
ENO3	1.08e-03	5.43e-04	0.00e+00	1.45e-03	2.36e-03	AFR, SAS
EPX	1.58e-03	1.22e-02	7.10e-03	1.04e-02	2.53e-02	NFE, FIN, AFR, SAS
ERCC3	1.66e-03	1.39e-03	1.46e-04	2.49e-03	8.88e-04	AFR
ERCC4	1.44e-03	4.39e-04	1.05e-04	1.58e-03	7.17e-04	AFR
EYS	1.71e-03	4.12e-03	1.12e-03	4.60e-03	4.09e-03	NFE, AFR, SAS
FAM161A	7.49e-04	2.29e-03	1.94e-04	4.98e-04	1.10e-03	NFE, SAS
FANCA	2.03e-03	1.69e-03	3.00e-03	1.49e-03	1.37e-03	FIN
FANCC	1.27e-03	1.59e-03	0.00e+00	1.45e-03	1.06e-03	NFE, AFR
FANCD2	1.57e-03	2.58e-03	2.92e-03	1.34e-03	1.13e-03	NFE, FIN
FANCM	1.97e-03	6.90e-03	1.07e-02	1.99e-03	3.81e-03	NFE, FIN, AFR, SAS
FASTKD2	9.84e-04	5.11e-04	1.66e-03	2.50e-04	5.83e-04	FIN
FKRP	8.31e-04	1.12e-04	8.79e-04	4.15e-05	2.19e-04	FIN
FMO3	9.44e-04	3.07e-03	4.86e-05	5.81e-04	1.88e-03	NFE, SAS
FOLR1	8.81e-04	6.98e-03	1.41e-03	1.04e-03	6.83e-05	NFE, FIN, AFR
FOXRED1	1.27e-03	7.53e-04	1.00e-04	4.16e-04	1.68e-03	SAS
FTCD	1.22e-03	7.53e-04	2.71e-04	1.41e-02	8.95e-04	AFR
FTO	1.34e-03	2.69e-04	1.94e-04	3.33e-04	1.37e-03	SAS
GAA	1.54e-03	7.93e-04	8.31e-04	7.06e-03	1.24e-03	AFR
GALC	1.48e-03	1.03e-03	1.61e-03	5.42e-04	7.24e-04	FIN
GALNT3	1.29e-03	4.44e-04	3.89e-04	5.52e-03	3.43e-04	AFR
GAMT	6.91e-04	7.38e-04	0.00e+00	4.15e-04	1.02e-04	NFE
GBE1	1.38e-03	4.98e-03	2.54e-03	8.70e-04	6.03e-04	NFE, FIN
GNPTG	1.03e-03	1.05e-03	1.46e-04	6.64e-04	4.13e-04	NFE
GP1BB	3.55e-04	1.33e-05	0.00e+00	0.00e+00	1.08e-03	SAS
GPX1	3.57e-04	5.03e-05	4.45e-04	4.16e-04	1.03e-04	FIN, AFR
GRHPR	9.48e-04	6.54e-04	1.90e-03	8.72e-04	5.13e-04	FIN
GRM6	1.36e-03	1.37e-03	3.58e-03	6.22e-04	1.81e-03	NFE, FIN, SAS
GYS2	1.58e-03	2.61e-03	2.92e-04	9.16e-04	8.16e-03	NFE, SAS
HAX1	9.09e-04	3.07e-04	3.89e-04	1.16e-03	2.73e-04	AFR
HEXA	1.34e-03	2.99e-03	3.40e-04	1.09e-03	4.10e-04	NFE
HGSNAT	1.44e-03	6.50e-04	5.02e-05	1.83e-03	4.58e-04	AFR
HMGCS2	1.32e-03	7.20e-04	0.00e+00	2.20e-03	1.60e-03	AFR, SAS
HOGA1	5.98e-04	2.77e-04	0.00e+00	5.39e-04	7.87e-04	SAS
IDUA	1.22e-03	2.55e-03	6.66e-03	4.15e-04	2.08e-04	NFE, FIN
IGHMBP2	1.69e-03	2.26e-03	9.30e-04	7.47e-04	1.68e-03	NFE
IL12RB1	1.53e-03	2.25e-03	3.96e-04	1.29e-03	1.28e-03	NFE
IRAK4	1.05e-03	2.85e-03	4.88e-04	8.30e-04	3.43e-04	NFE
ITGA7	1.88e-03	1.97e-03	4.42e-04	1.33e-03	3.25e-03	NFE, SAS
KCNV2	8.58e-04	9.31e-04	2.47e-04	1.45e-03	1.13e-03	NFE, AFR, SAS

Continued on next page

bioRxiv preprint doi: https://doi.org/10.64898/2025.12.05.692665; this version posted December 9, 2025. The copyright holder for this preprint (which was not certified by peer review) is the author/funder, who has granted bioRxiv a license to display the preprint in perpetuity. It is made available under a CC-BY 4.0 International license.

Gene	Square Root Mutational Target Size	Non-Finnish pPath	Finnish pPath	African pPath	South Asian pPath	Potentially Recessive Populations
KHK	9.71e-04	7.27e-04	4.87e-05	4.14e-05	1.84e-03	SAS
KLK4	6.63e-04	2.24e-04	0.00e+00	7.00e-03	2.08e-04	AFR
KLKB1	1.24e-03	8.84e-04	9.76e-05	6.64e-04	2.02e-03	SAS
KRT8	9.33e-04	1.46e-03	3.40e-04	4.57e-04	3.42e-04	NFE
LAMA2	3.09e-03	2.59e-03	9.78e-04	2.84e-03	3.21e-03	SAS
LMBRD1	1.33e-03	4.02e-04	4.86e-05	2.45e-03	7.63e-04	AFR
LOXHD1	2.34e-03	5.87e-03	4.41e-03	2.34e-03	2.48e-03	NFE, FIN, AFR, SAS
LPIN1	1.58e-03	6.40e-04	1.46e-04	1.80e-03	9.77e-04	AFR
LTC4S	5.97e-04	9.41e-03	4.97e-03	1.29e-03	4.23e-03	NFE, FIN, AFR, SAS
MAN2B1	1.62e-03	1.30e-03	3.56e-03	3.02e-03	1.20e-03	FIN, AFR
MARVELD2	1.28e-03	1.72e-03	9.77e-05	7.88e-04	1.91e-03	NFE, SAS
MASP2	1.21e-03	8.00e-04	2.45e-04	1.04e-03	1.24e-03	SAS
MCEE	5.54e-04	1.79e-03	4.87e-04	2.49e-04	0.00e+00	NFE
MMAA	1.21e-03	2.06e-03	3.40e-04	4.99e-04	1.48e-03	NFE, SAS
MMAB	7.71e-04	5.01e-04	5.27e-05	1.66e-04	9.56e-04	SAS
MOCS2	5.59e-04	9.76e-05	1.56e-03	8.29e-05	6.83e-05	FIN
MPV17	7.27e-04	9.49e-05	4.86e-05	2.61e-03	2.05e-04	AFR
MPZL2	8.33e-04	7.06e-04	4.86e-05	7.47e-04	2.49e-03	SAS
MS4A1	6.33e-04	1.14e-04	0.00e+00	9.53e-04	4.44e-04	AFR
MUTYH	1.23e-03	8.25e-04	1.46e-04	7.88e-04	6.86e-03	SAS
MYO15A	1.60e-03	3.37e-03	9.69e-04	3.33e-03	3.33e-03	NFE, AFR, SAS
NBN	1.24e-03	6.01e-04	6.80e-04	9.58e-04	1.33e-03	SAS
NDRG1	1.21e-03	3.40e-04	1.71e-03	8.30e-05	3.13e-04	FIN
NDUFAF2	6.55e-04	7.37e-04	0.00e+00	6.35e-04	2.07e-04	NFE
NDUFAF3	6.79e-04	5.53e-05	0.00e+00	4.14e-05	2.15e-03	SAS
NDUFAF5	9.39e-04	9.96e-04	9.76e-05	2.08e-04	7.56e-04	NFE
NDUFAF6	1.02e-03	4.34e-04	3.90e-04	4.60e-04	2.63e-03	SAS
NEB	3.12e-03	6.16e-03	3.88e-03	3.27e-03	3.51e-03	NFE, FIN, AFR, SAS
NME8	1.20e-03	1.05e-03	2.43e-04	1.34e-02	1.85e-03	AFR, SAS
NPC2	6.62e-04	5.38e-04	8.50e-03	3.98e-03	4.99e-03	FIN, AFR, SAS
NPHP3	1.41e-03	1.70e-03	1.02e-03	7.49e-04	7.54e-04	NFE
NPHP4	2.01e-03	1.24e-03	2.96e-04	1.95e-03	2.69e-03	SAS
NPHS1	1.84e-03	2.04e-03	3.45e-03	7.89e-04	1.09e-03	NFE, FIN
NT5E	1.09e-03	6.45e-04	0.00e+00	1.20e-03	4.45e-04	AFR
OBSL1	1.83e-03	9.70e-03	1.39e-02	2.74e-03	1.48e-03	NFE, FIN, AFR
OCA2	1.42e-03	3.75e-04	0.00e+00	2.74e-03	2.39e-04	AFR
OTOA	1.04e-03	1.79e-03	2.93e-04	3.96e-03	4.24e-03	NFE, AFR, SAS
OTOF	2.41e-03	4.53e-03	6.91e-04	2.90e-03	2.61e-03	NFE, AFR, SAS
P3H1	1.45e-03	5.57e-04	2.91e-04	7.95e-03	7.85e-04	AFR
PAH	1.27e-03	5.63e-03	3.40e-04	9.98e-04	9.22e-04	NFE
PCBD1	4.77e-04	8.56e-04	0.00e+00	2.07e-04	3.41e-05	NFE
PCK2	1.49e-03	2.50e-02	1.18e-03	4.36e-03	6.01e-03	NFE, AFR, SAS
PCNT	2.84e-03	2.56e-03	7.84e-04	4.44e-03	3.56e-03	AFR, SAS
PDE6A	1.54e-03	6.86e-04	1.95e-04	1.74e-03	1.81e-03	AFR, SAS
PEX7	1.00e-03	3.20e-03	4.37e-04	1.08e-03	2.05e-04	NFE, AFR
PHKB	1.86e-03	6.69e-03	1.94e-04	1.25e-03	4.53e-03	NFE, SAS
PHYH	8.32e-04	2.93e-04	1.31e-03	4.17e-04	1.37e-04	FIN
PKHD1	3.05e-03	2.18e-03	4.38e-04	2.50e-03	4.45e-03	SAS
PLOD2	1.57e-03	5.88e-04	6.81e-03	3.78e-04	6.56e-04	FIN
PMM2	8.98e-04	9.82e-04	2.45e-04	2.50e-04	7.53e-04	NFE
PNKP	1.20e-03	8.14e-04	9.72e-05	8.33e-04	1.21e-03	SAS
PPT1	9.73e-04	2.35e-03	9.72e-05	6.23e-04	2.05e-04	NFE
PRSS12	1.53e-03	3.04e-03	1.17e-03	1.78e-03	4.98e-03	NFE, AFR, SAS
PYGL	1.59e-03	1.42e-03	3.07e-04	2.74e-03	1.16e-03	AFR
RAB23	9.06e-04	2.52e-03	4.37e-04	4.98e-04	1.03e-04	NFE
RPGRIP1	1.36e-03	1.15e-03	1.02e-03	1.72e-03	1.21e-03	AFR
RPGRIP1L	2.02e-03	1.28e-03	5.83e-04	2.37e-03	1.71e-03	AFR
RSPH4A	1.17e-03	9.94e-04	3.89e-04	2.91e-04	1.33e-03	SAS
SGSH	1.01e-03	3.16e-04	1.12e-03	3.32e-04	1.03e-04	FIN
SLC10A2	7.84e-04	1.63e-03	0.00e+00	1.33e-03	2.06e-04	NFE, AFR
SLC12A3	1.63e-03	3.31e-03	1.48e-04	1.46e-03	8.57e-04	NFE
SLC22A12	1.18e-03	1.90e-04	1.04e-04	2.90e-04	2.60e-03	SAS

Continued on next page

bioRxiv preprint doi: https://doi.org/10.64898/2025.12.05.692665; this version posted December 9, 2025. The copyright holder for this preprint (which was not certified by peer review) is the author/funder, who has granted bioRxiv a license to display the preprint in perpetuity. It is made available under a CC-BY 4.0 International license.

Gene	Square Root Mutational Target Size	Non-Finnish pPath	Finnish pPath	African pPath	South Asian pPath	Potentially Recessive Populations
SLC25A13	1.56e-03	1.18e-03	0.00e+00	6.67e-04	2.43e-03	SAS
SLC25A20	1.10e-03	3.70e-04	4.86e-05	1.79e-03	2.73e-04	AFR
SLC29A3	8.50e-04	3.37e-04	4.86e-05	1.66e-04	2.01e-03	SAS
SLC34A3	1.03e-03	2.09e-03	5.46e-04	5.41e-04	1.06e-03	NFE, SAS
SLC6A19	1.38e-03	1.95e-03	4.67e-03	3.11e-03	1.74e-03	NFE, FIN, AFR, SAS
SLC7A7	1.08e-03	2.90e-04	1.33e-02	3.73e-04	8.53e-04	FIN
SMPD1	1.09e-03	4.51e-04	9.82e-05	1.24e-04	1.43e-03	SAS
SP110	8.81e-04	9.30e-04	1.94e-04	7.47e-04	4.92e-03	NFE, SAS
SPATA7	1.09e-03	7.09e-04	3.88e-04	1.08e-03	1.37e-03	SAS
SPINK5	1.74e-03	1.69e-03	1.90e-03	1.04e-03	1.20e-03	FIN
SRD5A3	7.35e-04	1.35e-04	4.99e-05	1.66e-04	3.02e-03	SAS
STXBP2	1.17e-03	1.37e-03	9.74e-05	7.58e-04	7.56e-04	NFE
SUMF1	1.05e-03	1.50e-04	0.00e+00	1.12e-03	3.10e-04	AFR
TBX19	9.90e-04	4.40e-04	5.30e-03	4.56e-04	6.83e-05	FIN
TCIRG1	1.56e-03	1.76e-03	1.30e-03	3.75e-04	9.34e-04	NFE
TGM1	1.53e-03	2.81e-03	5.88e-04	9.95e-04	5.81e-04	NFE
TMC6	1.42e-03	4.25e-04	0.00e+00	1.97e-03	6.72e-04	AFR
TMEM67	1.75e-03	2.34e-03	3.90e-04	1.08e-03	1.24e-03	NFE
TMEM70	7.45e-04	9.52e-04	4.85e-05	2.91e-04	6.83e-05	NFE
TMPRSS15	1.57e-03	8.22e-03	2.43e-04	2.80e-03	3.32e-03	NFE, AFR, SAS
TPP1	1.21e-03	6.82e-03	6.81e-04	7.05e-04	2.39e-04	NFE
TRIOBP	2.69e-03	3.58e-03	1.24e-03	2.17e-03	1.24e-03	NFE
TRIP11	2.29e-03	2.18e-03	3.45e-03	9.13e-04	1.27e-03	FIN
TYMP	9.91e-04	1.22e-03	4.99e-05	4.15e-04	3.76e-04	NFE
TYRP1	1.03e-03	7.51e-04	2.92e-04	5.65e-03	5.80e-04	AFR
UNC13D	1.90e-03	1.60e-03	3.44e-04	4.17e-04	3.09e-03	SAS
UPB1	1.16e-03	8.13e-03	5.93e-03	1.78e-03	1.05e-02	NFE, FIN, AFR, SAS
UROC1	1.33e-03	6.52e-04	1.47e-04	1.54e-03	8.60e-04	AFR
USH1C	1.44e-03	1.95e-03	2.43e-04	9.30e-03	8.89e-04	NFE, AFR
USH2A	3.41e-03	4.27e-03	2.38e-03	4.11e-03	5.46e-03	NFE, AFR, SAS
WDR35	1.99e-03	1.59e-03	4.56e-03	3.36e-03	2.12e-03	FIN, AFR, SAS
WDR72	1.56e-03	9.97e-04	1.75e-03	1.09e-03	1.71e-03	FIN, SAS
WRN	1.78e-03	6.60e-03	4.43e-03	2.95e-03	1.27e-03	NFE, FIN, AFR
XDH	1.86e-03	4.90e-03	4.86e-04	1.74e-03	2.64e-03	NFE, SAS
XPA	9.76e-04	3.01e-04	9.25e-03	6.65e-04	2.73e-04	FIN

Table S5: Select Recessive Genes GO Enrichment:
Gene Ontology enrichment of recessive disease genes in the expected range for recessive mutation-selection balance based on pLoF in Non-Finnish European, Finnish, African, and South Asian populations. Each row identifies the enrichment of purely recessive genes in biological, molecular, and cellular functions and the significance of this enrichment. Background gene list is all recessive disease genes.

Population	Ontology	ID	Description	GeneRatio	BgRatio	p-value	p.adjust	qvalue	Count	geneID
nfe	CC	GO:0016324	apical plasma membrane	.16/94	45/796	1.53e-05	1.58e-03	1.45e-03	16	ABCC2/ABCG5/ABCG8/ ASPM/CD36/CUBN/ DUOX2/FOLR1/MARVELD2/ OTOA/SLC10A2/SLC12A3/ SLC34A3/SLC6A19/TCIRG1/ USH2A
nfe	CC	GO:0045177	apical part of cell	.17/94	50/796	1.59e-05	1.58e-03	1.45e-03	17	ABCC2/ABCG5/ABCG8/ ASPM/CD36/CUBN/ DUOX2/FOLR1/MARVELD2/ OTOA/SLC10A2/SLC12A3/ SLC34A3/SLC6A19/TCIRG1/ USH1C/USH2A
nfe	CC	GO:0098862	cluster of actin-based cell projections	.12/94	32/796	1.13e-04	7.44e-03	6.85e-03	12	BBS2/CD36/CUBN/ FOLR1/LOXHD1/MYO15A/ SLC34A3/SLC6A19/TMPRSS15/ TRIOBP/USH1C/USH2A
nfe	CC	GO:0032420	stereocilium	.6/94	10/796	3.30e-04	1.64e-02	1.50e-02	6	BBS2/LOXHD1/MYO15A/ TRIOBP/USH1C/USH2A
nfe	CC	GO:0042995	cell projection	.31/94	154/796	5.76e-04	2.28e-02	2.10e-02	31	ALMS1/BBS2/CD36/ CEP290/CERKL/CNGA1/ CNGB1/CUBN/DNAAF1/ DNAH11/DNAI1/EYS/ FAM161A/FOLR1/GRM6/ IGHMBP2/LOXHD1/MYO15A/ NPHP3/NPHS1/OTOF/ PPT1/PRSS12/SLC10A2/ SLC34A3/SLC6A19/TMEM67/ TRIOBP/USH1C/USH2A/WRN
nfe	CC	GO:0098590	plasma membrane region	.22/94	97/796	8.65e-04	2.85e-02	2.62e-02	22	ABCC2/ABCG5/ABCG8/ ASPM/ATP7B/BBS2/ CD36/CNGA1/CNGB1/ CUBN/DUOX2/FOLR1/ MARVELD2/OTOA/OTOF/ SLC10A2/SLC12A3/SLC34A3/ SLC6A19/TCIRG1/TMEM67/ USH2A

Continued on next page

bioRxiv preprint doi: https://doi.org/10.64898/2025.12.05.692665; this version posted December 9, 2025. The copyright holder for this preprint (which was not certified by peer review) is the author/funder, who has granted bioRxiv a license to display the preprint in perpetuity. It is made available under a CC-BY 4.0 International license.

Population	Ontology	ID	Description	GeneRatio	BgRatio	p-value	p.adjust	qvalue	Count	geneID
nfe	CC	GO:0032421	stereocilium bundle	.6/94	12/796	1.19e-03	3.37e-02	3.10e-02	6	BBS2/LOXHD1/MYO15A/ TRIOBP/USH1C/USH2A
nfe	CC	GO:0098858	actin-based cell projection	.8/94	21/796	1.56e-03	3.77e-02	3.47e-02	8	BBS2/CUBN/LOXHD1/MYO15A/SLC10A2/TRIOBP/USH1C/USH2A
nfe	CC	GO:0120025	plasma membrane bounded cell projection	.29/94	149/796	1.71e-03	3.77e-02	3.47e-02	29	ALMS1/BBS2/CD36/CEP290/CERKL/CNGA1/CNGB1/CUBN/DNAAF1/DNAH11/DNAI1/EYS/FAM161A/FOLR1/GRM6/IGHMBP2/LOXHD1/MYO15A/NPHP3/PPT1/PRSS12/SLC10A2/SLC34A3/SLC6A19/TMEM67/TRIOBP/USH1C/USH2A/WRN
nfe	MF	GO:0016462	pyrophosphatase activity	.10/92	26/783	3.35e-04	2.01e-02	1.95e-02	10	ABCG5/ABCG8/APTX/ATP7B/BLM/FANCM/IGHMBP2/MMAA/RAB23/WRN
nfe	MF	GO:0016817	hydrolase activity, acting on acid anhydrides	.10/92	26/783	3.35e-04	2.01e-02	1.95e-02	10	ABCG5/ABCG8/APTX/ATP7B/BLM/FANCM/IGHMBP2/MMAA/RAB23/WRN
nfe	MF	GO:0016818	hydrolase activity, acting on acid anhydrides, in phosphorus-containing anhydrides	.10/92	26/783	3.35e-04	2.01e-02	1.95e-02	10	ABCG5/ABCG8/APTX/ATP7B/BLM/FANCM/IGHMBP2/MMAA/RAB23/WRN

bioRxiv preprint doi: https://doi.org/10.64898/2025.12.05.692665; this version posted December 9, 2025. The copyright holder for this preprint (which was not certified by peer review) is the author/funder, who has granted bioRxiv a license to display the preprint in perpetuity. It is made available under a CC-BY 4.0 International license.

bioRxiv preprint doi: https://doi.org/10.64898/2025.12.05.692665; this version posted December 9, 2025. The copyright holder for this preprint (which was not certified by peer review) is the author/funder, who has granted bioRxiv a license to display the preprint in perpetuity. It is made available under a CC-BY 4.0 International license.

Appendix

Appendix A: Theoretical results

Here we derive our approximate formula for mutation-selection-drift balance in the purely recessive case in the presence of inbreeding. We also discuss how it generalizes previous results and discuss various computational considerations.

One of our main results in the course of deriving this theorem is rigorously showing that a discrete-time Wright-Fisher model with strength of selection s_{hom} against homozygotes and inbreeding controlled by a rate of identity-by-descent, φ, converges to the same Wright-Fisher diffusion as a the standard Wright-Fisher model *without inbreeding* but with an effective population size of $N/(1+\varphi)$, an effective strength of selection against *heterozygotes* of φs_{hom}, and an effective strength of selection against homozygotes of $(1+\varphi)s_{hom}$.

A discrete-time Wright-Fisher model with inbreeding

We start by considering a single-locus biallelic discrete-time Wright-Fisher model. We fix the population size to be N diploids, and we label the two alleles "0" and "1". Suppose that the frequency of the "1" allele in generation t is f_t. The next generation is formed by first creating an infinitely large pool of gametes, where proportion f_t are the "1" allele. Then, each gamete independently mutates to the other allele with probability μ. We then consider pairing gametes to create zygotes. To model inbreeding, with probability φ a single gamete is pulled from the mutated gamete pool, and the two gametes in a zygote are identical to this chosen gamete. Otherwise, with probability $(1-\varphi)$ the two gametes are independently chosen from the mutated gamete pool. We then model purely recessive selection by removing zygotes homozygous for the "1" allele from the pool of zygotes with probability s_{hom}. Finally, the next generation is formed by randomly choosing N of these zygotes from the pool. This process defines a Markov chain on the frequency of the "1" allele. The goal of this section is to approximately compute the mean of this frequency at equilibrium.

From the discrete-time Wright-Fisher model to a Wright-Fisher diffusion process

Our first step will be to move from this unwieldy discrete-time Wright-Fisher model to a more analytically tractable diffusion. Following [95, Chapter 15.1], to prove that this process converges to a diffusion, we need to compute $\mathbb{E}[f_t - f_{t-1}|f_{t-1}]$ and $\mathbb{E}[(f_t - f_{t-1})^2|f_{t-1}]$ and show that both are $O(1/N)$, and we need to prove that $\mathbb{E}[(f_t - f_{t-1})^4|f_{t-1}]$ is $o(1/N)$. To calculate these quantities, we first introduce the notation $N_t^{(00)}, N_t^{(01)}, N_t^{(11)}$ for the number of individuals in generation t who are homozygous for the "0" allele, heterozygous, and homozygous for the "1" allele respectively. Under our model, the number of "1" alleles, $2Nf_t$ can be decomposed as $2Nf_t = 2N_t^{(11)} + N_t^{(01)}$.

bioRxiv preprint doi: https://doi.org/10.64898/2025.12.05.692665; this version posted December 9, 2025. The copyright holder for this preprint (which was not certified by peer review) is the author/funder, who has granted bioRxiv a license to display the preprint in perpetuity. It is made available under a CC-BY 4.0 International license.

Then, by construction we have that

$$\left(N_t^{(00)}, N_t^{(01)}, N_t^{(11)}\right) | f_{t-1} \sim \text{Multinomial}\Bigg(N, \frac{(1-\varphi)(1-\widetilde{f}_{t-1})^2 + \varphi(1-\widetilde{f}_{t-1})}{1 - s_{hom}\left[(1-\varphi)\widetilde{f}_{t-1}^2 + \varphi\widetilde{f}_{t-1}\right]}, \frac{2(1-\varphi)\widetilde{f}_{t-1}(1-\widetilde{f}_{t-1})}{1 - s_{hom}\left[(1-\varphi)\widetilde{f}_{t-1}^2 + \varphi\widetilde{f}_{t-1}\right]}, \frac{(1-s_{hom})\left[(1-\varphi)\widetilde{f}_{t-1}^2 + \varphi\widetilde{f}_{t-1}\right]}{1 - s_{hom}\left[(1-\varphi)\widetilde{f}_{t-1}^2 + \varphi\widetilde{f}_{t-1}\right]}\Bigg),$$

where

$$\widetilde{f}_{t-1} := f_{t-1} - 2\mu f_{t-1} + \mu$$

represents the frequency of the "1" allele in the gamete pool following mutation. Throughout we will assume — as is standard in population genetics — that μ and s_{hom} are $O(1/N)$. Given that later we will apply our results to even the homozygous lethal case ($s_{hom} = 1$), we will revisit this assumption when numerically checking the accuracy of our approximations.

Therefore,

$$\begin{aligned}\mathbb{E}\left[f_t - f_{t-1} \mid f_{t-1}\right] &= \frac{1}{2N}\mathbb{E}\left[N_t^{(01)} + 2N_t^{(11)} \mid f_{t-1}\right] - f_{t-1} \\ &= \frac{(1-\varphi)\widetilde{f}_{t-1}(1-\widetilde{f}_{t-1}) + (1-s_{hom})\left[(1-\varphi)\widetilde{f}_{t-1}^2 + \varphi\widetilde{f}_{t-1}\right]}{1 - s_{hom}\left[(1-\varphi)\widetilde{f}_{t-1}^2 + \varphi\widetilde{f}_{t-1}\right]} - f_{t-1},\end{aligned}$$

which after much algebra simplifies to

$$\mathbb{E}\left[f_t - f_{t-1} \mid f_{t-1}\right] = 1 + \frac{\widetilde{f}_{t-1} - 1}{1 - s_{hom}\left[(1-\varphi)\widetilde{f}_{t-1}^2 + \varphi\widetilde{f}_{t-1}\right]} - f_{t-1}.$$

Using the series expansion $1/(1-x) = \sum_{k=0}^{\infty} x^k$, which is convergent for $x \in (-1, 1)$, we obtain

$$\begin{aligned}\mathbb{E}\left[f_t - f_{t-1} \mid f_{t-1}\right] &= \widetilde{f}_{t-1} - f_{t-1} + s_{hom}\left[(1-\varphi)f_{t-1}^2 + \varphi f_{t-1}\right](f_{t-1} - 1) + O\left(\frac{1}{N^2}\right) \\ &= \mu(1-2f_{t-1}) + s_{hom}\left[(1-\varphi)f_{t-1}^3 - (1-2\varphi)f_{t-1}^2 - \varphi f_{t-1}\right] \\ &\quad + O\left(\frac{1}{N^2}\right) \\ &= \mu(1-2f_{t-1}) - s_{hom}(1+\varphi)f_{t-1}(1-f_{t-1})\left[f_{t-1} + \frac{\varphi}{\varphi+1}(1-2f_{t-1})\right] \\ &\quad + O\left(\frac{1}{N^2}\right)\end{aligned}$$

where in the first line we used that $\widetilde{f}_{t-1} = f_{t-1} + O(1/N)$. The slightly strange form of the final line shows that this expected change is equivalent (up to lower

bioRxiv preprint doi: https://doi.org/10.64898/2025.12.05.692665; this version posted December 9, 2025. The copyright holder for this preprint (which was not certified by peer review) is the author/funder, who has granted bioRxiv a license to display the preprint in perpetuity. It is made available under a CC-BY 4.0 International license.

order terms) to a Wright-Fisher model with an *effective* strength of selection against heterozygotes, $s_{het}^{(\text{eff})} := \varphi s_{hom}$, and an *effective* strength of selection against homozygotes of $s_{hom}^{(\text{eff})} := (1+\varphi)s_{hom}$. Overall, this is $O(1/N)$ because of our assumptions on μ and s_{hom}, and therefore the first moment of the change in allele frequency is consistent with convergence to a diffusion.

Similarly, using that for $(X_1, X_2, \ldots) \sim \text{Multinomial}(N, p_1, p_2, \ldots)$,

$$\mathbb{E}[(X_i + 2X_j)^2] - (\mathbb{E}[X_i + 2X_j])^2 = Np_i(1-p_i) + 4Np_j(1-p_j) - 4Np_ip_j,$$

we can derive

$$\begin{aligned}
\mathbb{E}\left[(f_t - f_{t-1})^2 \mid f_{t-1}\right] &= \mathbb{E}\left[\left(f_t - \mathbb{E}[f_t|f_{t-1}] + \mathbb{E}[f_t|f_{t-1}] - f_{t-1}\right)^2 \mid f_{t-1}\right] \\
&= \mathbb{E}\left[f_t^2|f_{t-1}\right] - \mathbb{E}\left[f_t|f_{t-1}\right]^2 + O\left(\frac{1}{N^2}\right) \\
&= \frac{1}{4N^2}\left(\mathbb{E}\left[\left(N_t^{(01)} + 2N_t^{(11)}\right)^2 |f_{t-1}\right] - \mathbb{E}\left[N_t^{(01)} + 2N_t^{(11)}|f_{t-1}\right]^2\right) \\
&\quad + O\left(\frac{1}{N^2}\right) \\
&= \frac{(1+\varphi)f_{t-1}(1-f_{t-1})}{2N} + O\left(\frac{1}{N^2}\right),
\end{aligned}$$

where the second equality relied on our result that the mean change in allele frequency is $O(1/N)$, and to obtain the final equality we used similar algebra and arguments as in the case of the first moment. This is a factor of $(1+\varphi)$ larger than the second moment of the allele frequency change under a standard Wright-Fisher diffusion, indicating that genetic drift is $(1+\varphi)$ times stronger than in a population without inbreeding.

Finally, we can show that the fourth moment is small by considering:

$$\begin{aligned}
\mathbb{E}\left[(f_t - f_{t-1})^4 \mid f_{t-1}\right] &= \mathbb{E}\left[\left((f_t - \mathbb{E}[f_t \mid f_{t-1}]) + (\mathbb{E}[f_t \mid f_{t-1}] - f_{t-1})\right)^4 \mid f_{t-1}\right] \\
&\leq 8\mathbb{E}\left[(f_t - \mathbb{E}[f_t \mid f_{t-1}])^4 \mid f_{t-1}\right] + 8\left(\mathbb{E}[f_t \mid f_{t-1}] - f_{t-1}\right)^4
\end{aligned}$$

where the inequality can be derived from Young's inequality or via convexity of $f(x) : x \mapsto x^4$. Continuing, we see

$$\begin{aligned}
&= \frac{1}{2N^4}\mathbb{E}\Bigg[\Big(N_t^{(01)} - \mathbb{E}[N_t^{(01)}|f_{t-1}] \\
&\qquad + 2N_t^{(11)} - \mathbb{E}[2N_t^{(11)}|f_{t-1}]\Big)^4 \Bigg| f_{t-1}\Bigg] \\
&\quad + O\left(\frac{1}{N^4}\right).
\end{aligned}$$

bioRxiv preprint doi: https://doi.org/10.64898/2025.12.05.692665; this version posted December 9, 2025. The copyright holder for this preprint (which was not certified by peer review) is the author/funder, who has granted bioRxiv a license to display the preprint in perpetuity. It is made available under a CC-BY 4.0 International license.

Applying Young's inequality again, we obtain

$$
\begin{aligned}
&\leq \frac{4}{N^4}\mathbb{E}\left[\left(N_t^{(01)} - \mathbb{E}[N_t^{(01)}|f_{t-1}]\right)^4 \middle| f_{t-1}\right] \\
&\quad + \frac{4}{N^4}\mathbb{E}\left[\left(2N_t^{(11)} - \mathbb{E}[2N_t^{(11)}|f_{t-1}]\right)^4\right] \\
&\quad + O\left(\frac{1}{N^4}\right) \\
&= O\left(\frac{1}{N^2}\right),
\end{aligned}
$$

where the final line follows because the fourth central moment of a Binomial(N, p) random variable is $O(N^2)$. This shows that the fourth moment of the change in allele frequencies is $o(1/N)$ as required to converge to a diffusion.

With these results in hand, we can now use the results of [95, Chapter 15.1] to show that in the large N limit this converges to a diffusion. In theory, this involves scaling time by a factor of N and keeping $N\mu$ and Ns_{hom} fixed as N gets large. Here, we will (somewhat) abuse notation, by doing this scaling and taking the limit to obtain the diffusion and then immediately undoing the scaling so that we retain μ and s_{hom} in interpretable units. This results in the population frequency satisfying the following stochastic differential equation (SDE):

$$
\begin{aligned}
df_t = &\left\{\mu(1-2f_t) - s_{hom}(1+\varphi)f_t(1-f_t)\left[f_t + \frac{\varphi}{\varphi+1}(1-2f_t)\right]\right\}dt \\
&+ \sqrt{\frac{(1+\varphi)}{2N}(f_t(1-f_t))}dW_t,
\end{aligned}
$$

where W_t is a Wiener process (Brownian motion).

Stationary distribution of Wright-Fisher diffusion

The advantage of having obtained this SDE is that we may now readily derive the equilibrium probability density function. First, we can appeal to the Kolmogorov forward equation (Fokker-Plank equation) to transform the SDE into a partial differential equation for the probability density function, which we will denote by $p(f, t)$. In particular, the Kolmogorov forward equation applied to our SDE implies

$$
\begin{aligned}
\frac{\partial}{\partial t}p(f,t) = &-\frac{\partial}{\partial f}\left[\left\{\mu(1-2f) - s_{hom}(1+\varphi)f(1-f)\left[f + \frac{\varphi}{\varphi+1}(1-2f)\right]\right\}p(f,t)\right] \\
&+ \frac{\partial^2}{\partial f^2}\left[\frac{(1+\varphi)f(1-f)}{4N}p(f,t)\right].
\end{aligned}
$$

At equilibrium, there should be no change in the density with respect to time. We will denote the equilibrium density by $p(f)$. Equating the PDE with zero and

bioRxiv preprint doi: https://doi.org/10.64898/2025.12.05.692665; this version posted December 9, 2025. The copyright holder for this preprint (which was not certified by peer review) is the author/funder, who has granted bioRxiv a license to display the preprint in perpetuity. It is made available under a CC-BY 4.0 International license.

integrating with respect to f results in

$$\begin{aligned}0 = &\frac{\mathrm{d}}{\mathrm{d}f}\left[\frac{(1+\varphi)f(1-f)}{4N}p(f)\right]\\ &-\left\{\mu(1-2f) - s_{hom}(1+\varphi)f(1-f)\left[f+\frac{\varphi}{\varphi+1}(1-2f)\right]\right\}p(f)\\ &+c,\end{aligned}$$

for some integration constant c, which can be shown to be zero by an argument about the "flux" of probability. Essentially, one can interpret the right hand side of the previous equation without the constant as the amount of probability mass "flowing through" the point f. Since the process is at equilibrium and exists on a bounded domain, and mass cannot "flow" past the boundaries, this implies that $c = 0$. Using this and that $\mathrm{d}/\mathrm{d}f[p(f)]/p(f) = \mathrm{d}/\mathrm{d}f \log p(f)$, and rearranging we arrive at

$$\begin{aligned}\frac{\mathrm{d}}{\mathrm{d}f}\log p(f) = &\frac{4N\mu(1-2f)}{(1+\varphi)f(1-f)}\\ &-4Ns_{hom}\left[f+\frac{\varphi}{1+\varphi}(1-2f)\right]\\ &-\frac{\mathrm{d}}{\mathrm{d}f}\log\left(\frac{(1+\varphi)f(1-f)}{4N}\right).\end{aligned}$$

This can now be readily solved up to the normalizing constant by direct integration, resulting in

$$p(f) \propto [f(1-f)]^{4N\mu/(1+\varphi)-1}\exp\left\{-4Ns_{hom}\frac{\varphi}{1+\varphi}f - 2Ns_{hom}\frac{1-\varphi}{1+\varphi}f^2\right\}.$$

A simplification under the assumption of strong selection

Unfortunately, obtaining the normalizing constant for this density is intractable. To make progress, we assume that Ns_{hom} is large so that f is small with very high probability. In this case, we drop the $(1-f)$ term, and instead of assuming that $f \in [0,1]$, we instead allow any $f \geq 0$, with the understanding that the probability that $f > 1$ is vanishingly small. This is intimately related to previous work and has connections to branching processes [96, 97]. In any case, ultimately we obtain

$$p(f) \propto f^{4N\mu/(1+\varphi)-1}\exp\left\{-4Ns_{hom}\frac{\varphi}{1+\varphi}f - 2Ns_{hom}\frac{1-\varphi}{1+\varphi}f^2\right\}.$$

Main analytical results and numerical considerations

Below, in Theorem 1 we prove a more general result that may be of independent interest that implies that at equilibrium the expected frequency under our simplified model is

$$\mathbb{E}[f] = \frac{\sum_{k=0}^{\infty}\frac{(-4Ns_{hom}\varphi/(1+\varphi)^k\Gamma(2N\mu/(1+\varphi)+(k+1)/2)}{k!(2Ns_{hom}(1-\varphi)/(1+\varphi))^{2N\mu/(1+\varphi)+(k+1)/2}}}{\sum_{\ell=0}^{\infty}\frac{(-4Ns_{hom}\varphi/(1+\varphi)^\ell\Gamma(2N\mu/(1+\varphi)+\ell/2)}{\ell!(2Ns_{hom}(1-\varphi)/(1+\varphi))^{2N\mu/(1+\varphi)+\ell/2}}}, \tag{S1}$$

bioRxiv preprint doi: https://doi.org/10.64898/2025.12.05.692665; this version posted December 9, 2025. The copyright holder for this preprint (which was not certified by peer review) is the author/funder, who has granted bioRxiv a license to display the preprint in perpetuity. It is made available under a CC-BY 4.0 International license.

where $\Gamma(\cdot)$ is the Gamma function.

In the limit of no inbreeding, this can be easily seen to reduce to

$$\lim_{\varphi \downarrow 0} \mathbb{E}[f] = \frac{\Gamma(2N\mu + 1/2)}{\Gamma(2N\mu)\sqrt{2Ns_{hom}}}.$$

Computing $\mathbb{E}[f]$ under our model with $\varphi = 0$ was one of the main results of [96], showing that our results are consistent with and generalize those.

The series appearing in the numerator and denominator of Equation S1 are convergent, and so this formula is "exact" (up to the diffusion and small f approximations needed to derive the density function in the first place). Unfortunately, the terms in the series can be astronomically large and have alternating signs. As a result, in our implementation of Equation S1, we use `mpmath` [98], a high precision library in python, and we compute all of the Gamma functions and factorials in log-space and perform cancellations before exponentiating. Furthermore, we typically truncate the series after a number of terms. We adaptively truncate the series by computing terms until the next term in the series is smaller in magnitude than the previous term and smaller than $\exp(-500)$.

Another downside of Equation S1 is that it provides little intuition into how the various parameters affect the equilibrium frequency without evaluating the equation. As a result, we also prove a general result, Theorem 2, about the large N limit. As we will show below, when φ is small, N must be quite large for this to be a good approximation (e.g., $N \gg 10{,}000$). In our case, this result implies that

$$\lim_{N \to \infty} \mathbb{E}[f] = -\frac{\varphi}{2(1-\varphi)} + \sqrt{\left(\frac{\varphi}{2(1-\varphi)}\right)^2 + \frac{\mu}{(1-\varphi)s_{hom}}}. \tag{S2}$$

This equation is much easier to reason about directly. For example, we see that when φ is 0, this reduces to $\sqrt{\mu/s_{hom}}$, the classic mutation selection balance result used in the main text. Similarly, when φ is large, this is well approximated by μ/s_{hom}, as can be seen via Taylor series. This is intuitive, because this would be the result of mutation selection balance if selection were acting on *heterozygotes* with strength s_{hom}. In essence, when inbreeding is strong, almost all individuals are homozygotes, and so we can envision the population as being essentially N haploids, and selection acts with strength s_{hom} to remove haploids with the deleterious allele. Mutations can occur on either of an individual's chromosomes, but both chromosome are equally likely to be passed on to their likely homozygous offspring. These factors of two cancel, resulting in the effective rate of mutation in this haploid model remaining μ.

Estimation of fraction of selection in heterozygotes

Lastly, we sought to estimate, for each recessive gene, what fraction of selective removal of pathogenic mutations occurs in heterozygotes, which we refer to as f_{het}, and defined:

$$f_{het} = \frac{2p_{path}(1-p_{path})s_{het}}{2p_{path}(1-p_{path})s_{het} + 2p_{path}^2 s_{hom}} \tag{S3}$$

For $p_{path} \geq \sqrt{\mu L}$ we set $\widehat{f_{het}} = 0$. Otherwise, after some basic cancellations from the expression above, we estimate $\widehat{f_{het}}$ under the conservative assumption that $s_{hom} = 1$, as

bioRxiv preprint doi: https://doi.org/10.64898/2025.12.05.692665; this version posted December 9, 2025. The copyright holder for this preprint (which was not certified by peer review) is the author/funder, who has granted bioRxiv a license to display the preprint in perpetuity. It is made available under a CC-BY 4.0 International license.

follows:

$$\begin{aligned} f_{het} &= \frac{(1-p_{path})s_{het}}{(1-p_{path})s_{het} + p_{path}s_{hom}} \\ &\geq \frac{(1-p_{path})s_{het}}{(1-p_{path})s_{het} + p_{path}} \end{aligned}$$

Noting that under mutation selection balance $2p(1-p)s_{het} = 2\mu L - 2p^2 s_{hom}$, and again setting $s_{hom} = 1$, we rearrange the latter expression to get:

$$\begin{aligned} f_{het} &\geq \frac{(1-p_{path})(\mu L - p_{path}^2)}{(1-p_{path})(\mu L - p_{path}^2) + p_{path}^2(1-p_{path})} \\ &= 1 - \frac{p_{path}^2}{\mu L}, \end{aligned} \tag{S4}$$

We use the latter as a lower-bound estimator for f_{het}.

Verification of approximations using `fastDTWF`

In addition to the comparisons to simulations presented in the main text, we also checked Equation S1 and Equation S2 against the equilibrium values obtained numerically using `fastDTWF` [99]. `fastDTWF` uses numerical approximations to compute the probability mass function of having different allele frequencies under geneal discrete-time Wright-Fisher models, including the one described at the beginning of this appendix. It uses principled numerical approximations, but does not perform any of the approximations to the model that we performed to obtain our main results (e.g., taking the diffusion limit or assuming that the frequencies are small with high probability).

The results are shown in Figures A1 to A3 where we vary N, μ, s_{hom}, and φ. Unless otherwise stated, we use a default value of $\mu = 10^{-5}$, $s_{hom} = 0.1$, and $\varphi = 0.02$. Overall, it is clear that as long as the product of N and s_{hom} is large enough, Equation S1 is extremely accurate even for fairly small N and large s_{hom}, where the assumptions we made during our derivation break down. For example, the results for $s_{hom} = 1$ are shockingly accurate given that our derivation required $s_{hom} = O(1/N)$.

bioRxiv preprint doi: https://doi.org/10.64898/2025.12.05.692665; this version posted December 9, 2025. The copyright holder for this preprint (which was not certified by peer review) is the author/funder, who has granted bioRxiv a license to display the preprint in perpetuity. It is made available under a CC-BY 4.0 International license.

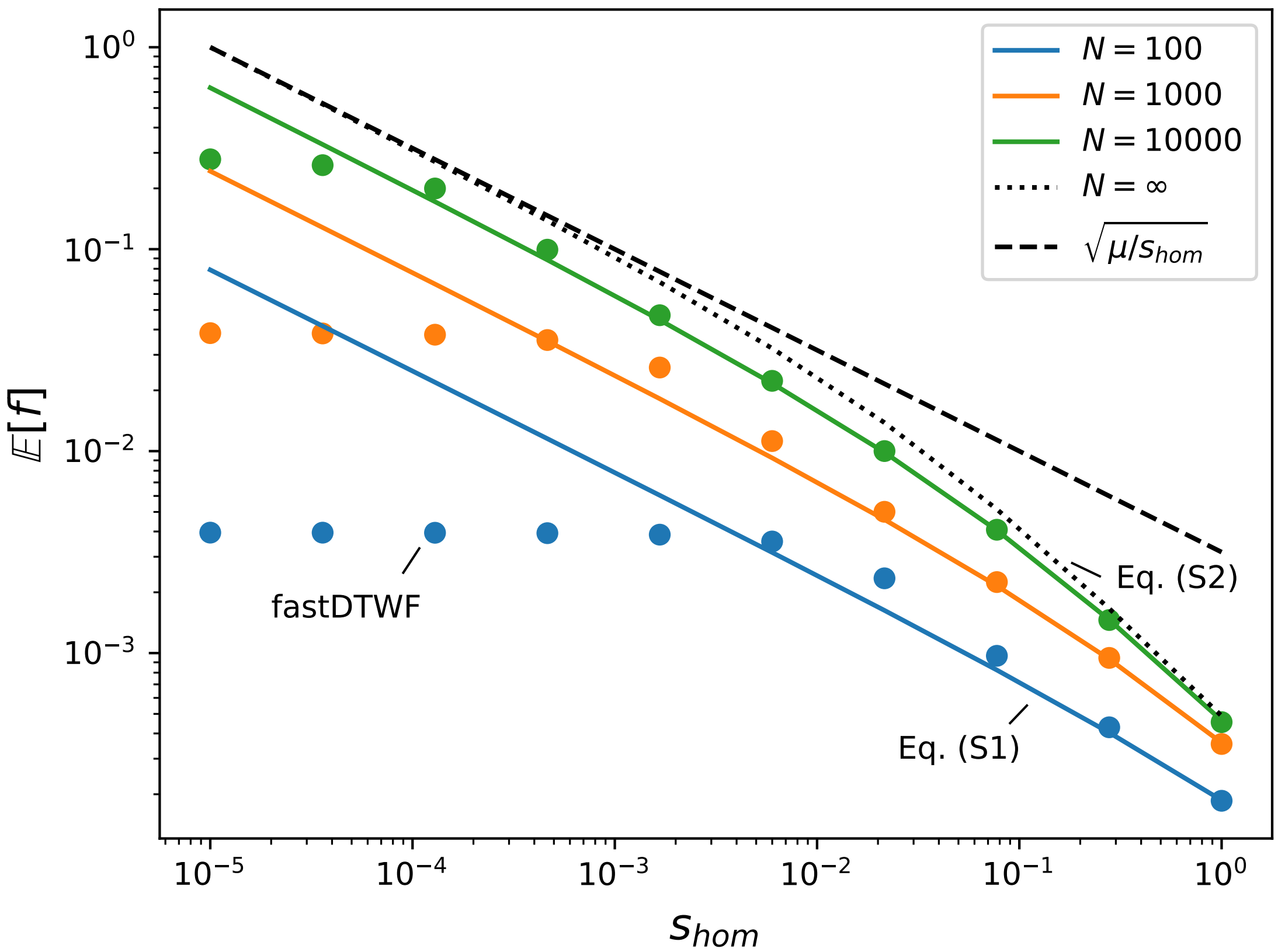

Figure A1: Comparison of `fastDTWF` with Equations S1 and S2, varying N and s_{hom}. The other parameters of the model are fixed at $\mu = 10^{-5}$ and $\varphi = 0.02$. `fastDTWF` is presented as dots and the results derived in this appendix are lines. The colored solid lines are from Equation S1, the dotted line is the $N \to \infty$ asymptotic result from Equation S2, and the dashed line is the asymptotic mutation-selection balance result without inbreeding, $\mathbb{E}[f] = \sqrt{\mu/s_{hom}}$.

bioRxiv preprint doi: https://doi.org/10.64898/2025.12.05.692665; this version posted December 9, 2025. The copyright holder for this preprint (which was not certified by peer review) is the author/funder, who has granted bioRxiv a license to display the preprint in perpetuity. It is made available under a CC-BY 4.0 International license.

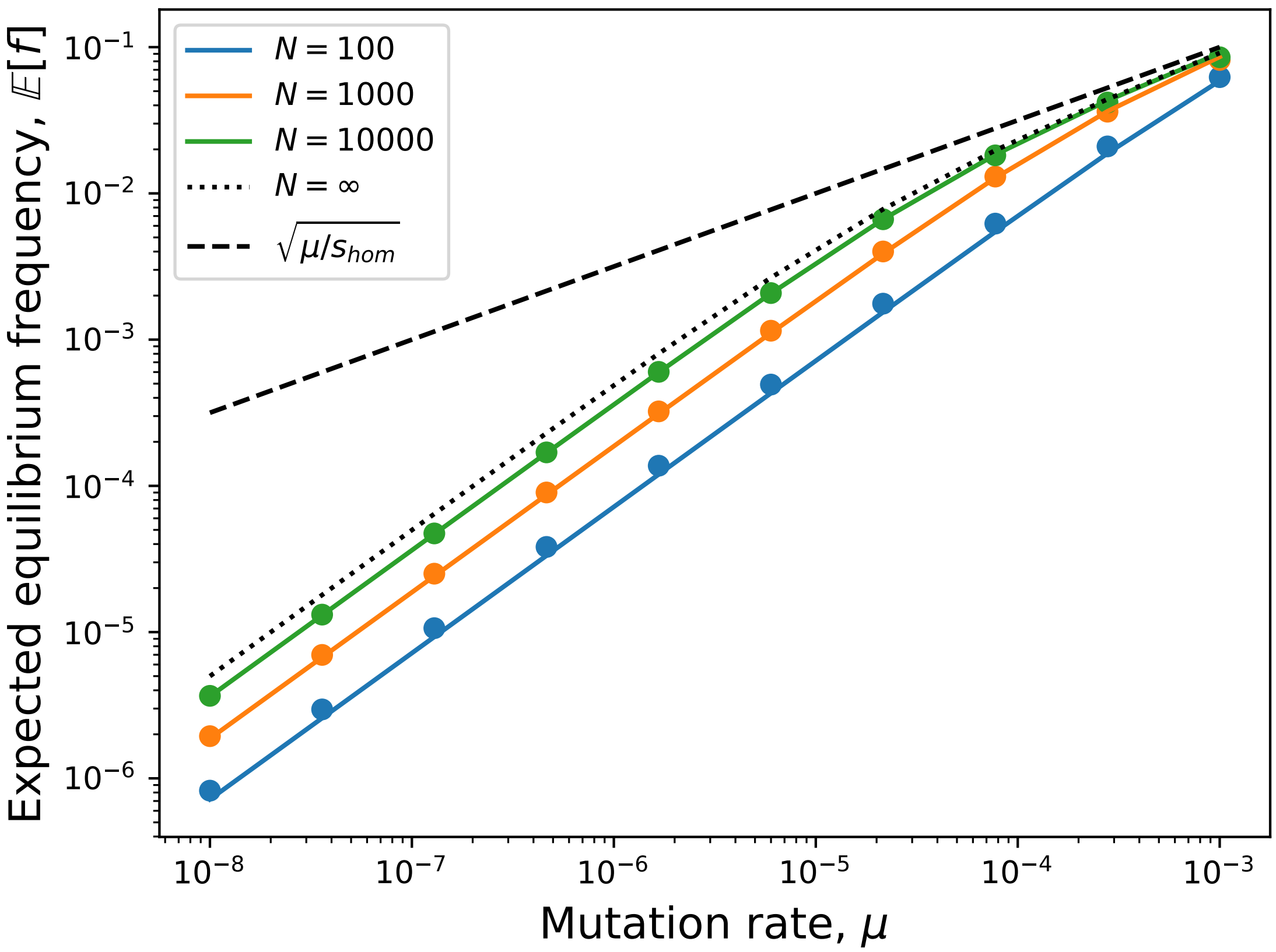

Figure A2: Comparison of `fastDTWF` with Equations S1 and S2, varying N and μ. The other parameters of the model are fixed at $s_{hom} = 0.1$ and $\varphi = 0.02$. `fastDTWF` is presented as dots and the results derived in this appendix are lines. The colored solid lines are from Equation S1, the dotted line is the $N \to \infty$ asymptotic result from Equation S2, and the dashed line is the asymptotic mutation-selection balance result without inbreeding, $\mathbb{E}[f] = \sqrt{\mu/s_{hom}}$.

bioRxiv preprint doi: https://doi.org/10.64898/2025.12.05.692665; this version posted December 9, 2025. The copyright holder for this preprint (which was not certified by peer review) is the author/funder, who has granted bioRxiv a license to display the preprint in perpetuity. It is made available under a CC-BY 4.0 International license.

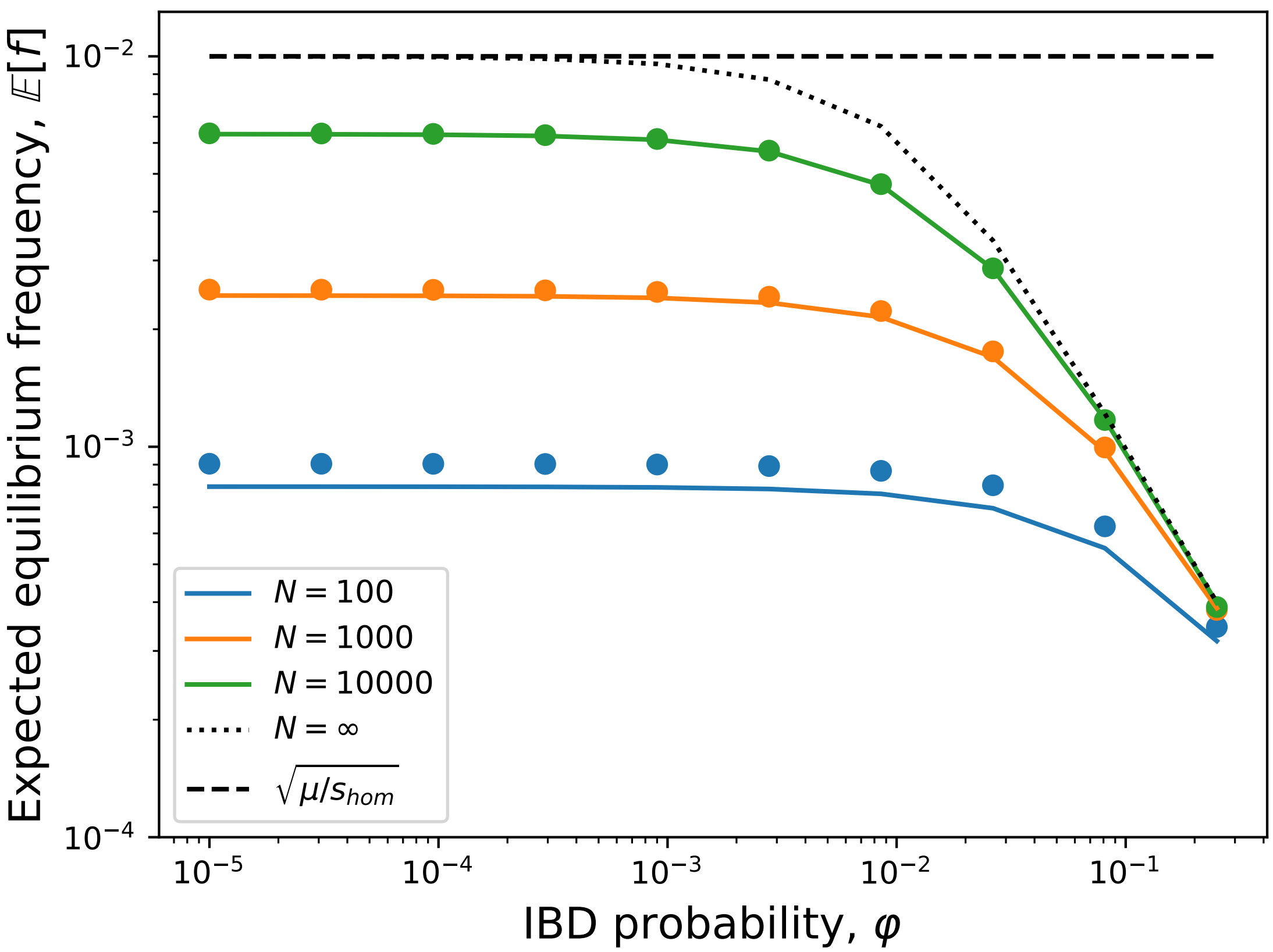

Figure A3: Comparison of `fastDTWF` with Equations S1 and S2, varying N and φ. The other parameters of the model are fixed at $s_{hom} = 0.1$ and $\mu = 10^{-5}$. `fastDTWF` is presented as dots and the results derived in this appendix are lines. The colored solid lines are from Equation S1, the dotted line is the $N \to \infty$ asymptotic result from Equation S2, and the dashed line is the asymptotic mutation-selection balance result without inbreeding, $\mathbb{E}[f] = \sqrt{\mu/s_{hom}}$.

bioRxiv preprint doi: https://doi.org/10.64898/2025.12.05.692665; this version posted December 9, 2025. The copyright holder for this preprint (which was not certified by peer review) is the author/funder, who has granted bioRxiv a license to display the preprint in perpetuity. It is made available under a CC-BY 4.0 International license.

Technical results

Here we prove our two technical results. The first theorem is fairly general and may be of independent interest, while the second Theorem is an easy application of Laplace's method to our setting.

Theorem 1. *Let X be a random variable supported on $[0, \infty)$ drawn from a distribution with probability density function*

$$p_X(x) \propto x^{a-1} \exp\left\{-bx - cx^2\right\},$$

for some $a > 0$, $b > 0$ and $c > 0$. Then,

$$\mathbb{E}[X] = \frac{\sum_{k=0}^{\infty} \frac{(-b)^k \Gamma\left(\frac{a}{2}+\frac{k}{2}+\frac{1}{2}\right)}{k! c^{a/2+k/2+1/2}}}{\sum_{\ell=0}^{\infty} \frac{(-b)^\ell \Gamma\left(\frac{a}{2}+\frac{\ell}{2}\right)}{\ell! c^{a/2+\ell/2}}},$$

where $\Gamma(\cdot)$ is the gamma function.

Proof. To begin, we note that

$$\mathbb{E}[X] = \frac{\int_0^\infty x^a \exp\left\{-bx - cx^2\right\} dx}{\int_0^\infty x^{a-1} \exp\left\{-bx - cx^2\right\} dx}. \tag{S5}$$

We will now obtain explicit formulas for the numerator and denominator. The approach for both is identical, so here we just derive the numerator. Substituting the series expansion of $\exp\{-bx\}$ into the numerator, we obtain

$$\begin{aligned}\int_0^\infty x^a \exp\left\{-bx - cx^2\right\} dx &= \int_0^\infty \sum_{k=0}^{\infty} \frac{(-b)^k}{k!} x^{a+k} \exp\left\{-cx^2\right\} dx \\ &= \sum_{k=0}^{\infty} \frac{(-b)^k}{k!} \int_0^\infty x^{a+k} \exp\left\{-cx^2\right\} dx.\end{aligned}$$

Changing the order of integration and summation on the second line requires justification, which we will provide at the end of this proof. At this point, we perform the change of variables $y = cx^2$ to obtain

$$\begin{aligned}\int_0^\infty x^a \exp\left\{-bx - cx^2\right\} dx &= \sum_{k=0}^{\infty} \frac{(-b)^k}{2k! c^{a/2+k/2+1/2}} \int_0^\infty y^{a/2+k/2-1/2} e^{-y} dy \\ &= \sum_{k=0}^{\infty} \frac{(-b)^k \Gamma\left(\frac{a}{2}+\frac{k}{2}+\frac{1}{2}\right)}{2k! c^{a/2+k/2+1/2}},\end{aligned}$$

where the final line follows by the integral definition of the gamma function. We can apply exactly the same logic to the denominator of the right hand side of Equation S5, substituting $a-1$ for a. Plugging the resulting series into Equation S5 and canceling factors of 2 proves the formula in the theorem.

To formally justify swapping the order of summations above, we appeal to Fubini's Theorem, which can be applied if the series is absolutely convergent. This follows from

bioRxiv preprint doi: https://doi.org/10.64898/2025.12.05.692665; this version posted December 9, 2025. The copyright holder for this preprint (which was not certified by peer review) is the author/funder, who has granted bioRxiv a license to display the preprint in perpetuity. It is made available under a CC-BY 4.0 International license.

noting

$$\int_0^\infty \sum_{k=0}^\infty \left| \frac{(-b)^k}{k!} x^{a+k} \exp\left\{-cx^2\right\} \right| dx = \int_0^\infty \sum_{k=0}^\infty \frac{b^k}{k!} x^{a+k} \exp\left\{-cx^2\right\} dx$$
$$= \int_0^\infty x^a \exp\left\{bx - cx^2\right\} dx.$$

To bound this integral, we note that $bx - cx^2 \leq -bx + b^2/c$ by concavity and considering the tangent at $x = b/c$. Therefore,

$$\int_0^\infty \sum_{k=0}^\infty \left| \frac{(-b)^k}{k!} x^{a+k} \exp\left\{-cx^2\right\} \right| dx \leq \exp\left\{b^2/c\right\} \int_0^\infty x^a \exp\left\{-bx\right\} dx$$
$$= \exp\left\{b^2/c\right\} \frac{\Gamma(a+1)}{b^{a+1}}$$
$$< \infty.$$

This also proves that the series appearing in the equation in the theorem are convergent. Additionally, the denominator is strictly positive, as can be seen by noting that the integrands in Equation S5 are strictly positive. As a result the series expression for $\mathbb{E}[X]$ is well-defined. □

Theorem 2. *Let X be a random variable supported on* $[0, \infty)$ *drawn from a distribution with probability density function*

$$p_X(x) \propto x^{Na-1} \exp\left\{-Nbx - Ncx^2\right\},$$

for some $a > 0$, $b > 0$ *and* $c > 0$. *Then, asymptotically as* $N \to \infty$,

$$\mathbb{E}[X] \sim \frac{-b + \sqrt{b^2 + 8ac}}{4c} + O\left(\frac{1}{N}\right).$$

Proof. As in Theorem 1, we begin by noting that

$$\mathbb{E}[X] = \frac{\int_0^\infty x^{Na} \exp\left\{-Nbx - Ncx^2\right\} dx}{\int_0^\infty x^{Na-1} \exp\left\{-Nbx - Ncx^2\right\} dx}. \tag{S6}$$

The proof is then a simple application of Laplace's method (e.g., [100, Chapter 6.4]) to the numerator and denominator of Equation S6, which proceeds as follows. To handle the numerator and denominator simultaneously, we will consider the exponent of x in the integrands to be $Na - \delta$ where $\delta = 0$ in the numerator and 1 in the denominator. We then rewrite the integral as

$$\int_0^\infty x^{Na-\delta} \exp\left\{-Nbx - Ncx^2\right\} dx = \int_0^\infty x^{-\delta} \exp\left\{N\left(a \log x - bx - cx^2\right)\right\} dx. \tag{S7}$$

Consider the function $\psi(x) := a \log x - bx - cx^2$. Computing the first two derivatives,

$$\psi'(x) = \frac{a}{x} - b - 2cx \tag{S8}$$
$$\psi''(x) = -\frac{a}{x^2} - 2c, \tag{S9}$$

bioRxiv preprint doi: https://doi.org/10.64898/2025.12.05.692665; this version posted December 9, 2025. The copyright holder for this preprint (which was not certified by peer review) is the author/funder, who has granted bioRxiv a license to display the preprint in perpetuity. It is made available under a CC-BY 4.0 International license.

we see by Equation S9 that ψ is concave on the relevant domain of x, and by setting Equation S8 to zero and solving, we see that ψ has a global maximum over $x > 0$ at $x_* := \frac{-b+\sqrt{b^2+8ac}}{4c}$.

Now, as $N \to \infty$ values of x away from x_* contribute exponentially less to the integral in Equation S7. We may therefore truncate the limits of integration to a small, but fixed region around x_* (say of width 2ϵ for some tiny ϵ that is constant in N) without changing the asymptotic value of the integral:

$$\int_0^\infty x^{-\delta} \exp\{N\psi(x)\}\, dx \sim \int_{x_*-\epsilon}^{x_*+\epsilon} x^{-\delta} \exp\{N\psi(x)\}\, dx.$$

This in turn, allows us to Taylor expand $x^{-\delta}$ and $\psi(x)$ about x_*:

$$\begin{aligned}\int_{x_*-\epsilon}^{x_*+\epsilon} & x^{-\delta} \exp\{N\psi(x)\}\, dx \\ &\sim \int_{x_*-\epsilon}^{x_*+\epsilon} x_*^{-\delta} \exp\left\{N\left(\psi(x_*) + \frac{1}{2}\psi''(x_*)(x-x_*)^2\right)\right\}\left(1+O\left(\frac{1}{N}\right)\right) dx,\end{aligned}$$

where the first order term $\psi'(x_*)(x - x_*)$ was dropped because it is zero as x_* is the maximizer of $\psi(x)$. We will explain why the error term is $O(1/N)$ at the end of the proof. Next, we again use the fact that the contributions to this integral outside of our window of width 2ϵ are asymptotically negligible, but this time we use this observation to expand our window of integration:

$$\begin{aligned}\int_{x_*-\epsilon}^{x_*+\epsilon} & x_*^{-\delta} \exp\left\{N\left(\psi(x_*) + \frac{1}{2}\psi''(x_*)(x-x_*)^2\right)\right\}\left(1+O\left(\frac{1}{N}\right)\right) dx \\ &\sim x_*^{-\delta} e^{N\psi(x_*)} \int_{-\infty}^{\infty} \exp\left\{\frac{1}{2}N\psi''(x_*)(x-x_*)^2\right\} dx \left(1+O\left(\frac{1}{N}\right)\right) \\ &= x_*^{-\delta} e^{N\psi(x_*)} \frac{\sqrt{2\pi}}{\sqrt{-N\psi''(x_*)}}\left(1+O\left(\frac{1}{N}\right)\right),\end{aligned}$$

where the final line follows from recognizing the integrand as the unnormalized density of Gaussian random variable with mean x_* and variance $-1/\psi''(x_*)$. The proof follows from substituting this expression with $\delta = 0$ and $\delta = 1$ into the numerator and denominator of Equation S6 respectively.

We now explain why the error term is $O(1/N)$. Further terms in the Taylor series for $\psi(x)$ can be collected into $\exp\{N(\psi'''(x_*)(x-x_*)^3 + \cdots)\}$, which can then itself be expanded as $1 + N(\psi'''(x_*)(x-x_*)^3/3! + \cdots) + \frac{1}{2}N^2(\psi'''(x_*)(x-x_*)^3/3! + \cdots)^2 + \cdots$. Multiplying this series with the Taylor series of $x^{-\delta}$ about x_* results in a series in powers of $(x - x_*)$. We will call the coefficient of $(x - x_*)^k$ in this expansion A_k, where we can see that $A_0 = x_*^{-\delta}$. Furthermore, A_2 is $O(1)$, A_4 is $O(N)$ and for $k \geq 6$, A_k is at

bioRxiv preprint doi: https://doi.org/10.64898/2025.12.05.692665; this version posted December 9, 2025. The copyright holder for this preprint (which was not certified by peer review) is the author/funder, who has granted bioRxiv a license to display the preprint in perpetuity. It is made available under a CC-BY 4.0 International license.

most $O(N^{k/3})$. We then have

$$\begin{aligned}\int_{x_*-\epsilon}^{x_*+\epsilon} & x^{-\delta} \exp\{N\psi(x)\}\,dx \\ &\sim e^{N\psi(x_*)} \int_{x_*-\epsilon}^{x_*+\epsilon} \sum_{k=0}^{\infty} A_k (x-x_*)^k \exp\left\{\frac{1}{2}N\psi''(x_*)(x-x_*)^2\right\} dx \\ &\sim e^{N\psi(x_*)} \sum_{k=0}^{\infty} A_k \int_{x_*-\epsilon}^{x_*+\epsilon} (x-x_*)^k \exp\left\{\frac{1}{2}N\psi''(x_*)(x-x_*)^2\right\} dx \\ &\sim e^{N\psi(x_*)} \sum_{k=0}^{\infty} A_k \int_{-\infty}^{\infty} (x-x_*)^k \exp\left\{\frac{1}{2}N\psi''(x_*)(x-x_*)^2\right\} dx.\end{aligned}$$

We then have that all odd order terms vanish because they are proportional to odd central moments of a Gaussian random variable. Similarly, the even terms are proportional to even central moments of a Gaussian with variance $O(1/N)$, and hence the integral involving $(x-x_*)^{2k}$ is $O(1/N^k)$. That is,

$$\begin{aligned}\int_{x_*-\epsilon}^{x_*+\epsilon} x^{-\delta} \exp\{N\psi(x)\}\,dx &\sim e^{N\psi(x_*)} \frac{\sqrt{2\pi}}{\sqrt{-N\psi''(x_*)}} \left(x_*^{-\delta} + \sum_{k=1}^{\infty} A_{2k} \times O\left(\frac{1}{N^k}\right)\right) \\ &\sim e^{N\psi(x_*)} \frac{\sqrt{2\pi}}{\sqrt{-N\psi''(x_*)}} \left(x_*^{-\delta} + O\left(\frac{1}{N}\right)\right),\end{aligned}$$

where the final line follows from noting that the terms involving A_2 and A_4 are $\leq O(1/N)$ and all other terms are strictly lower order by our bound that $A_{2k} \leq O(N^{2k/3})$ for $k \geq 3$, and hence $A_{2k} \times O(1/N^k) \leq O(N^{-k/3}) \leq O(1/N)$ for $k \geq 3$. □
